# Interventions to support parents and carers of young people with mental health difficulties: a systematic review protocol

**DOI:** 10.1136/bmjopen-2023-073940

**Published:** 2023-06-09

**Authors:** Faith Martin, Dania Dahmash, Anthony Tsang, Sarah Glover, Charlie Duncan, Sarah L Halligan

**Affiliations:** 1 School of Psychology, Cardiff University, Cardiff, UK; 2 Coventry University, Coventry, UK; 3 King’s College London, London, UK; 4 Parental Minds Community Interest Company, Devon, UK; 5 British Association for Counselling and Psychotherapy, Lutterworth, UK; 6 Department of Psychology, University of Bath, Bath, UK

**Keywords:** MENTAL HEALTH, Systematic Review, PUBLIC HEALTH, Adolescent

## Abstract

**Introduction:**

Globally, 8%–14% of children and young people (CYP) have a diagnosable mental health condition, many of whom receive no formal interventions. Parents/carers of CYP experience stress and distress owing to the mental health difficulties encountered by their CYP due to the lack of resources and support. Currently, little is known about (1) the content of interventions developed to support parents/carers nor (2) how effective interventions are at improving parents’/carers’ well-being. The planned review aims to address these two gaps.

**Method and analysis:**

A systematic review will be conducted to identify any study that describes an intervention aiming at least in part to support parents/carers with the impact of CYP (5–18 years) mental health difficulties, and to review any randomised controlled trials (RCTs) of these interventions. The following databases will be searched: MEDLINE, PsycINFO, CINAHL, AMED, EMBASE, Web of Science Core Collection and Cochrane Library CENTRAL, without any limitations applied. Analysis of the content of interventions will be structured using the Template for Intervention Description and Replication checklist as a framework. The effect of any RCTs on parents’/carers’ outcomes (including well-being, satisfaction with parenting, mental health) will be extracted and assessed using the Cochrane Risk-of-Bias Tool. Data will be synthesised narratively, with meta-analysis of RCT results, if appropriate.

**Ethical consideration and dissemination:**

The protocol is approved by Coventry University Ethical Committee (reference number: P139611). Results will be shared in academic publications and in accessible formats using social media and public webinars.

**PROSPERO registration number:**

CRD42022344453.

**STRENGTHS AND LIMITATIONS OF THIS STUDY**

- Narrative analysis will be used to analysed the intervention components which is a major strength, providing insight into the details of what the interventions reviewed cover.
- Primary screening of the articles, data extraction and quality assessment will be performed independently by two persons with extensive experience in systematic review methodology, to minimise the probability of personal biases.
- The review follows a rigorous method and the results of the review will be reported in accordance with Preferred Reporting Items for Systematic Reviews and Meta-Analyses reporting guidelines.
- This systematic review protocol reduces the possibility of duplication due to the transparency of the methods and processes that will be used; in addition, it reduces possible biases and allows for peer review.
- In this study, databases in languages other than English (French, German, Chinese, etc) will not be searched or included. This limitation may cause language bias.

## Introduction

Mental health conditions among children and young people (CYP) are increasingly common: globally around 8% of 5–9 years and 14% of 10–18 years old have a diagnosable mental health condition.^1^ In the UK, approximately one in six school-aged children have a mental health problem.^2^ Demand is outstripping service provision, with lengthy waiting times and only around a quarter (27%) of CYP who need mental health support in the UK are receiving it.^3^ This may place greater pressure on families to manage the difficulties on their own. Therefore, parents and carers (hereafter ‘parents’) are often experiencing stress and distress in relation to their CYP’s mental health difficulties, including depression, managing stigma, anxiety and work and financial pressures.^4–6^

Policy recommendations include seeing parents as partners in their CYP’s care,^7^ however, little attention is paid to the parent’s own needs. The WHO’s (WHO) report describes the importance of supporting parents’ mental health, however, focuses on parents with pre-existing mental health conditions and new parents.^1^ There is a reference to the needs of parents of CYP with developmental delays or difficulties, highlighting that they are likely to experience high levels of distress. While clearly vital, there is a need to also support parents with the understandable distress of having a CYP with a mental health difficulty.

Interventions have been developed that include parents to address CYP mental health. Parent training, family therapy and parent-co-delivered/parent-led cognitive behavioural therapy all, to some extent, involve the parent.^8–10^ However, the focus is frequently not on the parents’ well-being, nor on addressing the impact of CYP mental health difficulties on the parent. The content of interventions (or elements of interventions) that specifically aim to support parents has not yet been identified or summarised. This is important, as identification of the content can support the evaluation of effectiveness, for example, interventions may be focusing on just one element of parental distress by providing information, and thus not necessarily achieving good outcomes. The content of the interventions can be compared with the needs that have been identified for parents/carers.^11^

Given the lack of policy guidelines and existing evidence synthesis, this systematic review protocol outlines the process for identifying literature which addresses the following research questions:

1. What is the content of interventions or intervention components that have been designed to address parents’ well-being in the context of CYP mental health difficulties.
2. How effective are these interventions in terms of improving parents’ well-being outcomes, family well-being and mental health service utilisation?

## Method and analysis

The study has been registered in the International Prospective Register of Systematic Reviews (PROSPERO: CRD42022344453). This protocol follows the Preferred Reporting Items for Systematic Reviews and Meta-Analyses (PRISMA) for Protocols 2015 guidelines.^12^ The completed systematic review will adhere to the reporting guidelines of PRISMA-2020.^13^

### Study selection criteria

#### Participants

The participants to be included in this review will be parents of CYP (aged 5–18) who have a diagnosis of a mental health condition. This age range reflects the upper limit of most CYP- specific mental health services and covers CYP before they have gone to university, therefore, are more likely to still be at home with parents, with the lower limit relating to the youngest age of onset for most conditions.^14^ To be included, the majority (>50%) of the CYP must be within that age range. The CYP must have at least one diagnosed mental health condition from depression, anxiety disorders, psychoses, oppositional defiant and other externalising disorders, labels of emerging personality disorders, eating disorders and attention deficit (hyperactive) disorders. ‘Parents’ covers all adults (aged 18 years and over) in a formal parenting role, including biological parents, step-parents, other relatives assuming a parenting role, non-biological and adoptive parents, foster carers and other adults in legal guardian roles. The review does not cover parents/carers of CYP with special educational needs, including autism spectrum, as there are existing reviews and services and needs are different.^15^ Where the focus is on CYP with mental health difficulties and within that includes less than 50% CYP with developmental/special educational needs, studies would be included. Study samples can be drawn from any setting, for example, supporting parents of CYP on waiting lists, or receiving care through voluntary or statutory services, providing that the CYP has a diagnosed condition.

#### Intervention

The intervention must include a component specifically designed to meet the parents’/carers’ needs—be that information, emotional or social support. Interventions that train parents/carers to provide therapy or behavioural management techniques, where the focus of the intervention is solely on changing CYP outcomes, will not be included. Interventions that include therapy for the child but also support for parents/carers will be included. However, for research question 2, the intervention needs not to be described in full in the study, however, must provide sufficient evidence (eg, brief description, reference to a study that does describe the intervention) of relevance to the review.

#### Comparison

This only applies to research question 2. The comparison can be with any control group, including treatment as usual or active controls.

#### Outcomes

For research question 2, outcomes must be measured using validated tools only. Relevant outcomes are parental mental health (eg, depression, anxiety, stress); parent–CYP relationships; satisfaction with parenting; parenting self-efficacy; family well-being or parent/carer health service utilisation. Search terms relating to the outcomes are used to identify studies for research question 1 and also, to identify interventions that address our outcomes of interest.

#### Study design

For research question 1, relating to the content of interventions, studies must describe an intervention with at least one component to support parents’/carers’ needs or well-being. Studies can be of any design and do not need to report any intervention outcomes. For research question 2, randomised controlled trials will be included and assessed following Cochrane Reviewing guidelines.^16^ We will also include a narrative synthesis of studies that describe an intervention and its early-stage testing (eg, feasibility trials, case series).

### Search strategy

The following bibliographic databases were searched: MEDLINE(EBSCO), PsycINFO (Ovid), CINAHL Ultimate (EBSCO), AMED(EBSCO), EMBASE(Ovid), Web of Science Core Collection, Cochrane Library databases including: Cochrane Database of Systematic Reviews, Cochrane Central Register of Controlled Trials, Database of Abstracts of Reviews of Effects, Health Technology Assessment Database and National Health Service (NHS) Economic Evaluation Database. No filters were applied to the search.

The reference lists of any identified systematic reviews will be screened for additional studies not captured by the database search. Supplementary searches will be performed in the form of forward and backward citations using Citationchaser to potentially identify additional studies.^17^

The search terms will include five blocks of terms, all combined with “AND”. These terms cover:

1. Parents or carers.
2. Children and young people.
3. Mental health problems.
4. Intervention or treatment or therapy or peer support psychotherapy or counselling or group therapy.
5. Outcomes of interest including stress, anxiety, depression, burnout, or parenting satisfaction.

A scoping search was conducted using the above listed-terms, in which (3 827 850) records were identified. This scoping search was used to refine search terms, particularly the use of subject headings. To manage the downloading of records and the workload thereafter, two complementary search strategies will be used (online supplemental files 1 and 2). The first search strategy will focus on studies relating to CYP anxiety and/or depression. This is owing to these conditions being very common and representing a large portion of the overall records from the scoping search. The second search strategy then will identify studies linked to all other included CYP mental health difficulties. All criteria for inclusion/exclusion and data extraction will be conducted in the same way—this split is merely to manage the search results rather than conduct separate reviews. (If search 1 identifies studies that extend beyond anxiety and or depression to other included conditions, they will be included during screening). The planned analysis includes considering results in relation to CYP difficulty, as well as examining similarities/differences in intervention content and efficacy across CYP difficulties.

10.1136/bmjopen-2023-073940.supp1 Supplementary data

10.1136/bmjopen-2023-073940.supp2 Supplementary data

### Patient and public involvement

The scope of the review has been defined through discussion with parents of CYP with mental health difficulties (including with coauthor SG’s group ‘Parental Minds’). This patient and public involvement (PPI) engagement helped to design the study, refine the scope and specify the protocol, including the exclusion of autistic spectrum disorders and development difficulties from this review.

During the review, we will consult with a group of parents/carers as needed on decisions made, for example, the relevance of interventions to parents’ needs and thus inclusion in the review. Initial synthesis and interpretation of findings will be shared with our PPI group for discussion and refinement. PPI involvement, particularly in writing interpretation and recommendations, is planned for the writing of all outputs, the design of non-academic papers and wider dissemination activities.

### Data collection and analysis

#### Selection process

Rayyan software will be used to manage records.^18 19^ This facilitates blind decisions for inclusion/exclusion. Inclusion/exclusion criteria will be reviewed by stakeholders. A two-step process will be used. First, all titles and abstracts will be screened against the inclusion/exclusion criteria by two reviewers independently. Second, reports will be obtained for short-listed records and inclusion/exclusion will be conducted by two reviewers independently. Any discrepancies at either step will be resolved by a third reviewer if needed.

#### Data extraction and quality appraisal

A data extraction form will be created and piloted by at least two reviewers. Data will be extracted by two reviewers (DD and AT) and all data will be checked by a third reviewer. Data will be extracted into an Excel sheet. Discrepancies will be resolved by discussion with a third reviewer. For both questions extracted data will include, study characteristics (author, study design, setting, aim of the study and sample size) and population characteristics (CYP age, parents/carers age, ethnicity and relationship to CYP).

For research question 1, detailed information about the types of interventions developed will be extracted independently by two reviewers using the Template for Intervention Description and Replication (TIDieR) checklist as the basis to assess the completeness of the descriptions, therefore, in relation to this research question, this also covers the quality appraisal.^20^ This checklist includes: (1) descriptions of needs addressed, (2) aims of the intervention, (3) intervention content, (4) materials used, (5) inclusion criteria, (6) number and duration of sessions, (7) who delivers content and how, (8) where/what modality, (9) personalisation or tailoring of the intervention and (10) underpinning theory or approach. Information about implementation, including structure and content of training and supervision, and resource requirements will be included. If information is not specifically provided in a study, we will consult protocol and intervention design reports, and contact authors for further information as necessary. Where studies describe interventions for parents/carers and children, data will be extracted relating to the intervention or components for parents/carers. Data will also be extracted relating to any proposed mechanisms of how the intervention may operate.

For research question 2, details of the participant characteristics, the intervention itself (covered in research question 1), comparison group(s), and details of outcomes (eg, validated measures of parent/carer mental health (depression, anxiety, stress); parent–CYP relationships; satisfaction with parenting; family well-being and parent/carer health service utilisation) will be extracted. CYP MH service utilisation will also be extracted, if reported in parent/carer only interventions, to elucidate any impact on this important service outcome. Comparisons between intervention and control follow-up data points will be extracted, with effect sizes, to indicate the impact of the intervention in comparison to control. Quality will be appraised using the Cochrane Risk of Bias Tool (version 2).^16^

### Data synthesis

For research question 1, the aim is to describe in detail and categorise approaches to interventions to support parents/carers, mapping these onto parent/carer needs and describing underpinning theoretical approaches. No established a priori framework to categorise these interventions exists. Content analysis will be used to synthesise the data,^21–23^ within each data field from the TIDieR checklist. For example, codes for the underpinning theories will be created. Two reviewers will independently analyse and code the data, with a discussion of disagreements taken to a third reviewer, parent/carer working group or wider stakeholder group if required. Content analysis of intervention details will be tabulated, capturing the frequency of inclusion of different intervention content and other intervention details. Where multiple studies describe the same intervention, extracted data will be synthesised into a single entry for that intervention. Findings will also be grouped by CYP diagnosis and compared across different diagnoses.

For research question 2, Cochrane systematic review of interventions methods will be followed.^16^ Mean differences will be used for continuous measures and for outcomes where different measures are used (eg, anxiety, depression), standardised mean differences. In the absence of significant heterogeneity, and if there are sufficient studies, meta-analysis will be considered using random effects models using RevMan V.5.^24^ This will be conducted where possible in relation to each outcome variable group (eg, parents’ depression, parents’ stress). In the presence of significant heterogeneity, a narrative synthesis approach will be followed using Cochrane guidance.^25^ If there are sufficient studies, stratified analyses will be conducted by CYP diagnoses, intervention type and active versus inactive comparators. Sensitivity analyses will be performed excluding studies at high risk of bias. Publication bias will be explored using funnel plots if there are sufficient studies. Identified as important in PPI discussions, one reviewer will review the summaries, and comment on the extent to which the specifically identified outcome measures are patient-centred. A vital element of the analysis will be to compare the outcomes of interventions in relation to different CYP mental health difficulties. Interventions that do not select participants based on CYP mental health difficulty will be compared with those for specific problems, as well as comparisons between specific CYP mental health difficulties. If possible, these subgroup analyses will comprise of meta-analyses of studies by CYP mental health difficulty, however, if meta-analyses are not possible (eg, if high levels of heterogeneity as measured by I^2^), a narrative synthesis will consider the potential relevance of CYP mental health difficulty type to on reported outcomes, and considering any reported cross-cutting factors such a risk to CYP and duration of CYP mental health difficulty. Subgroup analysis will also be considered to examine differences in relation to whether CYP is receiving or awaiting care; whether care is via statutory or non-statutory services; and by intervention type if there are clear differences in theoretical approaches to the interventions.

### Ethics and dissemination

This study was approved by Coventry University ethical committee, reference number: P139611.

Dissemination will use multiple channels such as YouTube and social media, in addition to traditional academic outputs. We will disseminate findings via all our collaborators and partners in the NHS and charitable sectors to use their networks to share the findings widely. We particularly seek to reach UK policy-makers and clinicians using forums such as the Future (NHS) Collaboration platform. Parents/carers involved in the project will be sent the final outputs via email links/hard copies.

## Supplementary Material

Reviewer comments

Author's manuscript
