# Supplementary material for: Interventions to support parents and carers of young people with mental health difficulties: a systematic review protocol
Source: BMJ Open. 2023 Jun 9;13(6):e073940. doi: 10.1136/bmjopen-2023-073940 (PMC10277097; doi:10.1136/bmjopen-2023-073940)
Supplement: Supplementary data [file bmjopen-2023-073940supp001.pdf]

Search terms used for depression and anxiety studies:

Database: Medline

Search	Term
S1 (Parent)	TI ((parent or parents or parental or mother or father or care*giver or guardian* or carer* or paternal or maternal)) OR AB ((parent or parents or parental or mother or ather or care*giver or guardian* or carer* or paternal or maternal)) OR MM ("Parents+")
S2 (children)	TI ((children or adolescent* or adolescence or youth* or child or teenager* or pediatric* or paediatric* or kid* or teen* or young person or young people or boy* or girl* or juvenile*)) OR AB ((children or adolescent* or adolescence or youth* or child or teenager* or pediatric* or paediatric* or kid* or teen* or young person or young people or boy* or girl* or juvenile*)) OR MH ("Child+") OR MM ("Adolescent")
S3 (Mental Health)	TI ((Anxiety or depression or depressive or “obsessive compulsive disorder” or “OCD” or phobia or phobic or mood disorder or anxiety disorder or panic disorder or agoraphobia or internalising problem* or internalising problem* or internalizing problem* or internalizing disorder*)) OR AB ((Anxiety or depression or depressive or “obsessive compulsive disorder” or “OCD” or phobia or phobic or mood disorder or anxiety disorder or panic disorder or agoraphobia or internalising problem* or internalising problem* or internalizing problem* or internalizing disorder*) OR (MH "Depressive Disorder") OR (MH "Depressive Disorder, Major") OR (MH "Depressive Disorder, Treatment-Resistant") OR (MH "Dysthymic Disorder") OR (MM "Anxiety Disorders+"))
S4 (Intervention)	TI (intervention or treatment or therapy or peer support or support group or trial or psychotherapy or group support or talking therapy or counselling or group therapy or group intervention or trauma informed) OR AB (intervention or treatment or therapy or peer support or support group or trial or psychotherapy or group support or talking therapy or counselling or group therapy or group intervention or trauma informed) OR (MH "Psychotherapy") OR (MH "Self-Help Groups") OR (MH "Peer Group")
S5 (outcome)	TX (stress or anxiety or depression or burnout or worry* or "parent* satisfaction*" or wellbeing or mental health)
S6	S1 AND S2 AND S3 AND S4 AND S5

Database: PsycINFO

Search	Term
S1 (Parent)	TI ((parent or parents or parental or mother or father or care*giver or guardian* or carer* or paternal or maternal)) OR AB ((parent or parents or parental or mother or ather or care*giver or guardian* or carer* or paternal or maternal)) OR MM ("Parents+")
S2 (children)	TI ((children or adolescent* or adolescence or youth* or child or teenager* or pediatric* or paediatric* or kid* or teen* or young person or young people or boy* or girl* or juvenile*)) OR AB ((children or adolescent* or adolescence or youth* or child or teenager* or pediatric* or paediatric* or kid* or teen* or young person or young people or boy* or girl* or juvenile*)) OR MH ("Child+") OR MM ("Adolescent")
S3 (Mental Health)	TI ((Anxiety or depression or depressive or “obsessive compulsive disorder” or “OCD” or phobia or phobic or mood disorder or anxiety disorder or panic disorder or agoraphobia or internalising problem* or internalising problem* or internalizing problem* or internalizing disorder*)) OR AB ((Anxiety or depression or depressive or “obsessive compulsive disorder” or “OCD” or phobia or phobic or

	mood disorder or anxiety disorder or panic disorder or agoraphobia or internalising problem* or internalising problem* or internalizing problem* or internalizing disorder*) OR (MH "Depressive Disorder") OR (MH "Depressive Disorder, Major") OR (MH "Depressive Disorder, Treatment-Resistant") OR (MH "Dysthymic Disorder") OR (MM "Anxiety Disorders+"))
S4 (Intervention)	TI (intervention or treatment or therapy or peer support or support group or trial or psychotherapy or group support or talking therapy or counselling or group therapy or group intervention or trauma informed) OR AB (intervention or treatment or therapy or peer support or support group or trial or psychotherapy or group support or talking therapy or counselling or group therapy or group intervention or trauma informed) OR (MH "Psychotherapy") OR (MH "Self-Help Groups") OR (MH "Peer Group")
S5 (outcome)	TX (stress or anxiety or depression or burnout or worry* or "parent* satisfaction*" or wellbeing or mental health)
S6	S1 AND S2 AND S3 AND S4 AND S5

Database: CINAHL ULTIMATE

**Search**	**Term**
S1 (Parent)	TI ((parent or parents or parental or mother or father or care*giver or guardian* or carer* or paternal or maternal)) OR AB ((parent or parents or parental or mother or ather or care*giver or guardian* or carer* or paternal or maternal)) OR MM ("Parents+")
S2 (children)	TI ((children or adolescent* or adolescence or youth* or child or teenager* or pediatric* or paediatric* or kid* or teen* or young person or young people or boy* or girl* or juvenile*)) OR AB ((children or adolescent* or adolescence or youth* or child or teenager* or pediatric* or paediatric* or kid* or teen* or young person or young people or boy* or girl* or juvenile*)) OR MH ("Child+") OR MM ("Adolescent")
S3 (Mental Health)	TI ((Anxiety or depression or depressive or “obsessive compulsive disorder” or “OCD” or phobia or phobic or mood disorder or anxiety disorder or panic disorder or agoraphobia or internalising problem* or internalising problem* or internalizing problem* or internalizing disorder*)) OR AB ((Anxiety or depression or depressive or “obsessive compulsive disorder” or “OCD” or phobia or phobic or mood disorder or anxiety disorder or panic disorder or agoraphobia or internalising problem* or internalising problem* or internalizing problem* or internalizing disorder*) OR (MH "Depressive Disorder") OR (MH "Depressive Disorder, Major") OR (MH "Depressive Disorder, Treatment-Resistant") OR (MH "Dysthymic Disorder") OR (MM "Anxiety Disorders+"))
S4 (Intervention)	TI (intervention or treatment or therapy or peer support or support group or trial or psychotherapy or group support or talking therapy or counselling or group therapy or group intervention or trauma informed) OR AB (intervention or treatment or therapy or peer support or support group or trial or psychotherapy or group support or talking therapy or counselling or group therapy or group intervention or trauma informed) OR (MH "Psychotherapy") OR (MH "Self-Help Groups") OR (MH "Peer Group")
S5 (outcome)	TX (stress or anxiety or depression or burnout or worry* or "parent* satisfaction*" or wellbeing or mental health)
S6	S1 AND S2 AND S3 AND S4 AND S5

Database: AMED

Search	Term
S1 (Parent)	TI ((parent or parents or parental or mother or father or care*giver or guardian* or carer* or paternal or maternal)) OR AB ((parent or parents or parental or mother or ather or care*giver or guardian* or carer* or paternal or maternal)) OR MM ("Parents+")
S2 (children)	TI ((children or adolescent* or adolescence or youth* or child or teenager* or pediatric* or paediatric* or kid* or teen* or young person or young people or boy* or girl* or juvenile*)) OR AB ((children or adolescent* or adolescence or youth* or child or teenager* or pediatric* or paediatric* or kid* or teen* or young person or young people or boy* or girl* or juvenile*)) OR MH ("Child+") OR MM ("Adolescent")
S3 (Mental Health)	TI ((Anxiety or depression or depressive or “obsessive compulsive disorder” or “OCD” or phobia or phobic or mood disorder or anxiety disorder or panic disorder or agoraphobia or internalising problem* or internalising problem* or internalizing problem* or internalizing disorder*)) OR AB ((Anxiety or depression or depressive or “obsessive compulsive disorder” or “OCD” or phobia or phobic or mood disorder or anxiety disorder or panic disorder or agoraphobia or internalising problem* or internalising problem* or internalizing problem* or internalizing disorder*) OR (MH "Depressive Disorder") OR (MH "Depressive Disorder, Major") OR (MH "Depressive Disorder, Treatment-Resistant") OR (MH "Dysthymic Disorder") OR (MM "Anxiety Disorders+"))
S4 (Intervention)	TI (intervention or treatment or therapy or peer support or support group or trial or psychotherapy or group support or talking therapy or counselling or group therapy or group intervention or trauma informed) OR AB (intervention or treatment or therapy or peer support or support group or trial or psychotherapy or group support or talking therapy or counselling or group therapy or group intervention or trauma informed) OR (MH "Psychotherapy") OR (MH "Self-Help Groups") OR (MH "Peer Group")
S5 (outcome)	TX (stress or anxiety or depression or burnout or worry* or "parent* satisfaction*" or wellbeing or mental health)
S7	S1 AND S2 AND S3 AND S4 AND S5

Database: The Cochrane Library (including the Cochrane Database of Systematic Reviews, the Cochrane Central Register of Controlled Trials (CENTRAL), the Database of Abstracts of Reviews of Effects, the Health Technology Assessment Database, and the NHS Economic Evaluation Database)

Search	Term
S1 (Parent)	TI ((parent or parents or parental or mother or father or care*giver or guardian* or carer* or paternal or maternal)) OR AB ((parent or parents or parental or mother or ather or care*giver or guardian* or carer* or paternal or maternal)) OR MM ("Parents+")
S2 (children)	TI ((children or adolescent* or adolescence or youth* or child or teenager* or pediatric* or paediatric* or kid* or teen* or young person or young people or boy* or girl* or juvenile*)) OR AB ((children or adolescent* or adolescence or youth* or child or teenager* or pediatric* or paediatric* or kid* or teen* or young person or young people or boy* or girl* or juvenile*)) OR MH ("Child+") OR MM ("Adolescent"))
S3 (Mental Health)	TI ((Anxiety or depression or depressive or “obsessive compulsive disorder” or “OCD” or phobia or phobic or mood disorder or anxiety disorder or panic disorder or agoraphobia or internalising problem* or internalising problem* or internalizing problem* or internalizing disorder*)) OR AB ((Anxiety or depression or

	depressive or "obsessive compulsive disorder" or "OCD" or phobia or phobic or mood disorder or anxiety disorder or panic disorder or agoraphobia or internalising problem* or internalising problem* or internalizing problem* or internalizing disorder*) OR (MH "Depressive Disorder") OR (MH "Depressive Disorder, Major") OR (MH "Depressive Disorder, Treatment-Resistant") OR (MH "Dysthymic Disorder") OR (MM "Anxiety Disorders+"))
S4 (Intervention)	TI (intervention or treatment or therapy or peer support or support group or trial or psychotherapy or group support or talking therapy or counselling or group therapy or group intervention or trauma informed) OR AB (intervention or treatment or therapy or peer support or support group or trial or psychotherapy or group support or talking therapy or counselling or group therapy or group intervention or trauma informed) OR (MH "Psychotherapy") OR (MH "Self-Help Groups") OR (MH "Peer Group")
S5 (outcome)	TX (stress or anxiety or depression or burnout or worry* or "parent* satisfaction*" or wellbeing or mental health)
S7	S1 AND S2 AND S3 AND S4 AND S5

Database: Web of Science Core Collection

Search	Terms
1	(TI=(parent OR parents OR parental OR mother OR father OR care*giver OR guardian* OR carer* OR paternal OR maternal)) OR (AB=(parent OR parents OR parental OR mother OR father OR care*giver OR guardian* OR carer* OR paternal OR maternal))
2	(TI=(children OR adolescent* OR adolescence OR youth* OR child OR teenager* OR pediatric* OR paediatric* OR kid* OR teen* OR 'young person' OR 'young people' OR boy* OR girl* OR juvenile*)) OR (AB=(children OR adolescent* OR adolescence OR youth* OR child OR teenager* OR pediatric* OR paediatric* OR kid* OR teen* OR 'young person' OR 'young people' OR boy* OR girl* OR juvenile*))
3	TI ((Anxiety or depression or depressive or "obsessive compulsive disorder" or "OCD" or phobia or phobic or mood disorder or anxiety disorder or panic disorder or agoraphobia or internalising problem* or internalising problem* or internalizing problem* or internalizing disorder*)) OR AB ((Anxiety or depression or depressive or "obsessive compulsive disorder" or "OCD" or phobia or phobic or mood disorder or anxiety disorder or panic disorder or agoraphobia or internalising problem* or internalising problem* or internalizing problem* or internalizing disorder*) OR (MH "Depressive Disorder") OR (MH "Depressive Disorder, Major") OR (MH "Depressive Disorder, Treatment-Resistant") OR (MH "Dysthymic Disorder") OR (MM "Anxiety Disorders+"))
4	(TI=(intervention OR treatment OR therapy OR 'peer support' OR 'support group' OR trial OR psychotherapy OR 'group support' OR 'talking therapy' OR counselling OR 'group therapy' OR 'group intervention' OR 'trauma informed' OR 'self help group' OR 'peer group')) OR (AB=(intervention OR treatment OR therapy OR 'peer support' OR 'support group' OR trial OR psychotherapy OR 'group support' OR 'talking therapy' OR counselling OR 'group therapy' OR 'group intervention' OR 'trauma informed' OR 'self help group' OR 'peer group'))
5	(TI=(stress OR anxiety OR depression OR burnout OR worry* OR 'parent* satisfaction*' OR wellbeing OR 'mental health')) OR (AB=(stress OR anxiety OR depression OR burnout OR worry* OR 'parent* satisfaction*' OR wellbeing OR 'mental health'))
6	#5 AND #4 AND #3 AND #2 AND #1

Database: EMBASE

Search	Terms
1	'parent'/exp OR parent OR 'parents'/exp OR parents OR parental OR 'mother'/exp OR mother OR 'father'/exp OR father OR care*giver OR guardian* OR carer* OR paternal OR 'maternal'/exp OR maternal:ti,ab
2	children OR adolescent* OR adolescence OR youth* OR child OR teenager* OR pediatric* OR paediatric* OR kid* OR teen* OR 'young person' OR 'young people' OR boy* OR girl* OR juvenile*:ti,ab
3	anxiety OR depression OR depressive OR 'obsessive compulsive disorder' OR 'ocd' OR phobia OR phobic OR 'mood disorder' OR 'anxiety disorder':ab,ti OR 'panic disorder' OR agoraphobia OR 'internalising problem*' OR 'internalising disorder*' OR 'internalizing problem*' OR 'internalizing disorder*':ab,ti
4	intervention OR treatment OR therapy OR 'peer support' OR 'support group' OR trial OR psychotherapy OR 'group support' OR 'talking therapy' OR counselling OR 'group therapy' OR 'group intervention' OR 'trauma informed' OR 'self help group' OR 'peer group':ti,ab
5	stress OR anxiety OR depression OR burnout OR worry* OR 'parent* satisfaction*' OR wellbeing OR 'mental health':ti,ab
6	#1 AND #2 AND #3 AND #4 AND #5
