# Supplementary material for: Interventions to support parents and carers of young people with mental health difficulties: a systematic review protocol
Source: BMJ Open. 2023 Jun 9;13(6):e073940. doi: 10.1136/bmjopen-2023-073940 (PMC10277097; doi:10.1136/bmjopen-2023-073940)
Supplement: Supplementary data [file bmjopen-2023-073940supp002.pdf]

Search terms used excluding depression and anxiety terms:

Database: Medline

Search	Term
S1	TI ((parent or parents or parental or mother or father or care*giver or guardian* or carer* or paternal or maternal)) OR AB ((parent or parents or parental or mother or ather or care*giver or guardian* or carer* or paternal or maternal)) OR MM ("Parents+")
S2	TI ((children or adolescent* or adolescence or youth* or child or teenager* or pediatric* or paediatric* or kid* or teen* or young person or young people or boy* or girl* or juvenile*)) OR AB ((children or adolescent* or adolescence or youth* or child or teenager* or pediatric* or paediatric* or kid* or teen* or young person or young people or boy* or girl* or juvenile*)) OR MH ("Child+") OR MM ("Adolescent")
S3	TI ((attention deficit disorder* or “attention deficit hyperactive disorder* “or “ADHD”) OR AB (attention deficit disorder* or “attention deficit hyperactive disorder* “or “ADHD”)OR MH (“Attention Deficit Disorder with Hyperactivity”) OR (TI ((Eating disorder* or eating problem*)) OR AB ((Eating disorder* or eating problem*) OR MH(“Feeding and Eating Disorders+”) OR (TI ((Emerging personality disorder* or emerging personality problem*)) OR AB ((Emerging personality disorder* or emerging personality problem*)) OR MH (“Personality Disorder+”)) OR (TI ((Externalising disorder* or externalising problem* or externalizing disorder* or externalizing problem*) OR AB (Externalising disorder* or externalising problem* or externalizing disorder* or externalizing problem*) OR TX (“Externalising disorder”)) OR (TI ((Oppositional defiant disorder* or oppositional defiant problem*)) OR AB ((Oppositional defiant disorder* or oppositional defiant problem*) OR (MH“Attention Deficit and Disruptive Behavior Disorders+”)) OR (TI ((Psychos* or psychotic disorder* or psychotic problem*)) OR AB ((Psychos* or psychotic disorder* or psychotic problem*)) OR MH (“Psychotic Disorders+)) OR (TI ((Anxiety or depression or depressive or “obsessive compulsive disorder” or “OCD” or phobia or phobic or mood disorder or anxiety disorder or panic disorder or agoraphobia or internalising problem* or internalising problem* or internalizing problem* or internalizing disorder*)) OR AB ((Anxiety or depression or depressive or “obsessive compulsive disorder” or “OCD” or phobia or phobic or mood disorder or anxiety disorder or panic disorder or agoraphobia or internalising problem* or internalising problem* or internalizing problem* or internalizing disorder*) OR (MH "Depressive Disorder") OR (MH "Depressive Disorder, Major") OR (MH "Depressive Disorder, Treatment-Resistant") OR (MH "Dysthymic Disorder") OR (MM "Anxiety Disorders+"))
S4	TI (intervention or treatment or therapy or peer support or support group or trial or psychotherapy or group support or talking therapy or counselling or group therapy or group intervention or trauma informed) OR AB (intervention or treatment or therapy or peer support or support group or trial or psychotherapy or group support or talking therapy or counselling or group therapy or group intervention or trauma informed) OR (MH "Psychotherapy") OR (MH "Self-Help Groups") OR (MH "Peer Group")
S5	TX (stress or anxiety or depression or burnout or worry* or "parent* satisfaction*" or wellbeing or mental health)
S6	TI ((Anxiety or depression or depressive or “obsessive compulsive disorder” or “OCD” or phobia or phobic or mood disorder or anxiety disorder or panic disorder or agoraphobia or internalising problem* or internalising problem* or internalizing problem* or internalizing disorder*)) OR AB ((Anxiety or depression or

	depressive or "obsessive compulsive disorder" or "OCD" or phobia or phobic or mood disorder or anxiety disorder or panic disorder or agoraphobia or internalising problem* or internalising problem* or internalizing problem* or internalizing disorder*) OR (MH "Depressive Disorder") OR (MH "Depressive Disorder, Major") OR (MH "Depressive Disorder, Treatment-Resistant") OR (MH "Dysthymic Disorder") OR (MM "Anxiety Disorders+"))
S6	S1 AND S2 AND S3 AND S4 AND S5 NOT S6

Database: PsycINFO

Search	Term
S1	TI ((parent or parents or parental or mother or father or care*giver or guardian* or carer* or paternal or maternal)) OR AB ((parent or parents or parental or mother or ather or care*giver or guardian* or carer* or paternal or maternal)) OR MM ("Parents+")
S2	TI ((children or adolescent* or adolescence or youth* or child or teenager* or pediatric* or paediatric* or kid* or teen* or young person or young people or boy* or girl* or juvenile*)) OR AB ((children or adolescent* or adolescence or youth* or child or teenager* or pediatric* or paediatric* or kid* or teen* or young person or young people or boy* or girl* or juvenile*)) OR MH ("Child+") OR MM ("Adolescent")
S3	TI ((attention deficit disorder* or "attention deficit hyperactive disorder* "or "ADHD") OR AB (attention deficit disorder* or "attention deficit hyperactive disorder* "or "ADHD")OR MH ("Attention Deficit Disorder with Hyperactivity") OR (TI ((Eating disorder* or eating problem*)) OR AB ((Eating disorder* or eating problem*) OR MH("Feeding and Eating Disorders+") OR (TI ((Emerging personality disorder* or emerging personality problem*)) OR AB ((Emerging personality disorder* or emerging personality problem*)) OR MH ("Personality Disorder+")) OR (TI ((Externalising disorder* or externalising problem* or externalizing disorder* or externalizing problem*) OR AB (Externalising disorder* or externalising problem* or externalizing disorder* or externalizing problem*) OR TX ("Externalising disorder")) OR (TI ((Oppositional defiant disorder* or oppositional defiant problem*)) OR AB ((Oppositional defiant disorder* or oppositional defiant problem*) OR (MH"Attention Deficit and Disruptive Behavior Disorders+")) OR (TI ((Psychos* or psychotic disorder* or psychotic problem*)) OR AB ((Psychos* or psychotic disorder* or psychotic problem*)) OR MH ("Psychotic Disorders+)) OR (TI ((Anxiety or depression or depressive or "obsessive compulsive disorder" or "OCD" or phobia or phobic or mood disorder or anxiety disorder or panic disorder or agoraphobia or internalising problem* or internalising problem* or internalizing problem* or internalizing disorder*)) OR AB ((Anxiety or depression or depressive or "obsessive compulsive disorder" or "OCD" or phobia or phobic or mood disorder or anxiety disorder or panic disorder or agoraphobia or internalising problem* or internalising problem* or internalizing problem* or internalizing disorder*) OR (MH "Depressive Disorder") OR (MH "Depressive Disorder, Major") OR (MH "Depressive Disorder, Treatment-Resistant") OR (MH "Dysthymic Disorder") OR (MM "Anxiety Disorders+")
S4	TI (intervention or treatment or therapy or peer support or support group or trial or psychotherapy or group support or talking therapy or counselling or group therapy or group intervention or trauma informed) OR AB (intervention or treatment or therapy or peer support or support group or trial or psychotherapy or group support or talking therapy or counselling or group therapy or group intervention or trauma informed) OR (MH "Psychotherapy") OR (MH "Self-Help Groups") OR (MH "Peer Group")

S5	TX (stress or anxiety or depression or burnout or worry* or "parent* satisfaction*" or wellbeing or mental health)
S6	TI ((Anxiety or depression or depressive or "obsessive compulsive disorder" or "OCD" or phobia or phobic or mood disorder or anxiety disorder or panic disorder or agoraphobia or internalising problem* or internalising problem* or internalizing problem* or internalizing disorder*)) OR AB ((Anxiety or depression or depressive or "obsessive compulsive disorder" or "OCD" or phobia or phobic or mood disorder or anxiety disorder or panic disorder or agoraphobia or internalising problem* or internalising problem* or internalizing problem* or internalizing disorder*) OR (MH "Depressive Disorder") OR (MH "Depressive Disorder, Major") OR (MH "Depressive Disorder, Treatment-Resistant") OR (MH "Dysthymic Disorder") OR (MM "Anxiety Disorders+"))
S7	S1 AND S2 AND S3 AND S4 AND S5 NOT S6

Database: CINAHL ULTIMATE

**Search**	**Term**
S1	TI ((parent or parents or parental or mother or father or care*giver or guardian* or carer* or paternal or maternal)) OR AB ((parent or parents or parental or mother or ather or care*giver or guardian* or carer* or paternal or maternal)) OR MM ("Parents+")
S2	TI ((children or adolescent* or adolescence or youth* or child or teenager* or pediatric* or paediatric* or kid* or teen* or young person or young people or boy* or girl* or juvenile*)) OR AB ((children or adolescent* or adolescence or youth* or child or teenager* or pediatric* or paediatric* or kid* or teen* or young person or young people or boy* or girl* or juvenile*)) OR MH ("Child+") OR MM ("Adolescent")
S3	TI ((attention deficit disorder* or "attention deficit hyperactive disorder* "or "ADHD") OR AB (attention deficit disorder* or "attention deficit hyperactive disorder* "or "ADHD")OR MH ("Attention Deficit Disorder with Hyperactivity") OR (TI ((Eating disorder* or eating problem*)) OR AB ((Eating disorder* or eating problem*) OR MH("Feeding and Eating Disorders+") OR (TI ((Emerging personality disorder* or emerging personality problem*)) OR AB ((Emerging personality disorder* or emerging personality problem*)) OR MH ("Personality Disorder+")) OR (TI ((Externalising disorder* or externalising problem* or externalizing disorder* or externalizing problem*) OR AB (Externalising disorder* or externalising problem* or externalizing disorder* or externalizing problem*) OR TX ("Externalising disorder")) OR (TI ((Oppositional defiant disorder* or oppositional defiant problem*)) OR AB ((Oppositional defiant disorder* or oppositional defiant problem*) OR (MH"Attention Deficit and Disruptive Behavior Disorders+")) OR (TI ((Psychos* or psychotic disorder* or psychotic problem*)) OR AB ((Psychos* or psychotic disorder* or psychotic problem*)) OR MH ("Psychotic Disorders+)) OR (TI ((Anxiety or depression or depressive or "obsessive compulsive disorder" or "OCD" or phobia or phobic or mood disorder or anxiety disorder or panic disorder or agoraphobia or internalising problem* or internalising problem* or internalizing problem* or internalizing disorder*)) OR AB ((Anxiety or depression or depressive or "obsessive compulsive disorder" or "OCD" or phobia or phobic or mood disorder or anxiety disorder or panic disorder or agoraphobia or internalising problem* or internalising problem* or internalizing problem* or internalizing disorder*) OR (MH "Depressive Disorder") OR (MH "Depressive Disorder, Major") OR (MH "Depressive Disorder, Treatment-Resistant") OR (MH "Dysthymic Disorder") OR (MM "Anxiety Disorders+")
S4	TI (intervention or treatment or therapy or peer support or support group or trial or psychotherapy or group support or talking therapy or counselling or group therapy

	or group intervention or trauma informed) OR AB (intervention or treatment or therapy or peer support or support group or trial or psychotherapy or group support or talking therapy or counselling or group therapy or group intervention or trauma informed) OR (MH "Psychotherapy") OR (MH "Self-Help Groups") OR (MH "Peer Group")
S5	TX (stress or anxiety or depression or burnout or worry* or "parent* satisfaction*" or wellbeing or mental health)
S6	TI ((Anxiety or depression or depressive or “obsessive compulsive disorder” or “OCD” or phobia or phobic or mood disorder or anxiety disorder or panic disorder or agoraphobia or internalising problem* or internalising problem* or internalizing problem* or internalizing disorder*)) OR AB ((Anxiety or depression or depressive or “obsessive compulsive disorder” or “OCD” or phobia or phobic or mood disorder or anxiety disorder or panic disorder or agoraphobia or internalising problem* or internalising problem* or internalizing problem* or internalizing disorder*) OR (MH "Depressive Disorder") OR (MH "Depressive Disorder, Major") OR (MH "Depressive Disorder, Treatment-Resistant") OR (MH "Dysthymic Disorder") OR (MM "Anxiety Disorders+"))
S6	S1 AND S2 AND S3 AND S4 AND S5 NOT S6

Database: AMED

**Search**	**Term**
S1	TI ((parent or parents or parental or mother or father or care*giver or guardian* or carer* or paternal or maternal)) OR AB ((parent or parents or parental or mother or ather or care*giver or guardian* or carer* or paternal or maternal)) OR MM ("Parents+")
S2	TI ((children or adolescent* or adolescence or youth* or child or teenager* or pediatric* or paediatric* or kid* or teen* or young person or young people or boy* or girl* or juvenile*)) OR AB ((children or adolescent* or adolescence or youth* or child or teenager* or pediatric* or paediatric* or kid* or teen* or young person or young people or boy* or girl* or juvenile*)) OR MH ("Child+") OR MM ("Adolescent")
S3	TI ((attention deficit disorder* or “attention deficit hyperactive disorder* “or “ADHD”) OR AB (attention deficit disorder* or “attention deficit hyperactive disorder* “or “ADHD”)OR MH (“Attention Deficit Disorder with Hyperactivity”) OR (TI ((Eating disorder* or eating problem*)) OR AB ((Eating disorder* or eating problem*) OR MH(“Feeding and Eating Disorders+”) OR (TI ((Emerging personality disorder* or emerging personality problem*)) OR AB ((Emerging personality disorder* or emerging personality problem*)) OR MH (“Personality Disorder+”)) OR (TI ((Externalising disorder* or externalising problem* or externalizing disorder* or externalizing problem*) OR AB (Externalising disorder* or externalising problem* or externalizing disorder* or externalizing problem*) OR TX (“Externalising disorder”)) OR (TI ((Oppositional defiant disorder* or oppositional defiant problem*)) OR AB ((Oppositional defiant disorder* or oppositional defiant problem*) OR (MH“Attention Deficit and Disruptive Behavior Disorders+”)) OR (TI ((Psychos* or psychotic disorder* or psychotic problem*)) OR AB ((Psychos* or psychotic disorder* or psychotic problem*)) OR MH (“Psychotic Disorders+)) OR (TI ((Anxiety or depression or depressive or “obsessive compulsive disorder” or “OCD” or phobia or phobic or mood disorder or anxiety disorder or panic disorder or agoraphobia or internalising problem* or internalising problem* or internalizing problem* or internalizing disorder*)) OR AB ((Anxiety or depression or depressive or “obsessive compulsive disorder” or “OCD” or phobia or phobic or mood disorder or anxiety disorder or panic disorder or agoraphobia or internalising problem* or internalising problem* or internalizing

	problem* or internalizing disorder*) OR (MH "Depressive Disorder") OR (MH "Depressive Disorder, Major") OR (MH "Depressive Disorder, Treatment-Resistant") OR (MH "Dysthymic Disorder") OR (MM "Anxiety Disorders+")
S4	TI (intervention or treatment or therapy or peer support or support group or trial or psychotherapy or group support or talking therapy or counselling or group therapy or group intervention or trauma informed) OR AB (intervention or treatment or therapy or peer support or support group or trial or psychotherapy or group support or talking therapy or counselling or group therapy or group intervention or trauma informed) OR (MH "Psychotherapy") OR (MH "Self-Help Groups") OR (MH "Peer Group")
S5	TX (stress or anxiety or depression or burnout or worry* or "parent* satisfaction*" or wellbeing or mental health)
S6	TI ((Anxiety or depression or depressive or “obsessive compulsive disorder” or “OCD” or phobia or phobic or mood disorder or anxiety disorder or panic disorder or agoraphobia or internalising problem* or internalising problem* or internalizing problem* or internalizing disorder*)) OR AB ((Anxiety or depression or depressive or “obsessive compulsive disorder” or “OCD” or phobia or phobic or mood disorder or anxiety disorder or panic disorder or agoraphobia or internalising problem* or internalising problem* or internalizing problem* or internalizing disorder*) OR (MH "Depressive Disorder") OR (MH "Depressive Disorder, Major") OR (MH "Depressive Disorder, Treatment-Resistant") OR (MH "Dysthymic Disorder") OR (MM "Anxiety Disorders+"))
S7	S1 AND S2 AND S3 AND S4 AND S5 NOT S6

Database: The Cochrane Library (including the Cochrane Database of Systematic Reviews, the Cochrane Central Register of Controlled Trials (CENTRAL), the Database of Abstracts of Reviews of Effects, the Health Technology Assessment Database, and the NHS Economic Evaluation Database)

Search	Term
S1	TI ((parent or parents or parental or mother or father or care*giver or guardian* or carer* or paternal or maternal)) OR AB ((parent or parents or parental or mother or ather or care*giver or guardian* or carer* or paternal or maternal)) OR MM ("Parents+")
S2	TI ((children or adolescent* or adolescence or youth* or child or teenager* or pediatric* or paediatric* or kid* or teen* or young person or young people or boy* or girl* or juvenile*)) OR AB ((children or adolescent* or adolescence or youth* or child or teenager* or pediatric* or paediatric* or kid* or teen* or young person or young people or boy* or girl* or juvenile*)) OR MH ("Child+") OR MM ("Adolescent"))
S3	attention deficit disorder* or attention deficit hyperactive disorder* or ADHD or Eating disorder* or eating problem* or Emerging personality disorder or emerging personality problem* or Externalising disorder* or externalising problem* or externalizing disorder* or externalizing problem* or Oppositional defiant disorder* or oppositional defiant problem* or Psychos* or psychotic disorder* or psychotic problem* or Anxiety or depression or depressive or obsessive compulsive disorder or OCD or phobia or phobic or mood disorder or anxiety disorder or panic disorder or agoraphobia or internalising problem* or internalising problem* or internalizing problem* or internalizing disorder*
S4	TI (intervention or treatment or therapy or peer support or support group or trial or psychotherapy or group support or talking therapy or counselling or group therapy or group intervention or trauma informed) OR AB (intervention or treatment or

	therapy or peer support or support group or trial or psychotherapy or group support or talking therapy or counselling or group therapy or group intervention or trauma informed) OR (MH "Psychotherapy") OR (MH "Self-Help Groups") OR (MH "Peer Group")
S5	TX (stress or anxiety or depression or burnout or worry* or "parent* satisfaction*" or wellbeing or mental health)
S6	anxiety OR depression OR depressive OR 'obsessive compulsive disorder' OR 'ocd' OR phobia OR phobic OR 'mood disorder' OR 'anxiety disorder':ab,ti OR 'panic disorder' OR agoraphobia OR 'internalising problem*' OR 'internalising disorder*' OR 'internalizing problem*' OR 'internalizing disorder*':ab,ti
S7	S1 AND S2 AND S3 AND S4 AND S5 NOT S6

Database: Web of Science Core Collection

Search	Terms
1	(TI=(parent OR parents OR parental OR mother OR father OR care*giver OR guardian* OR carer* OR paternal OR maternal)) OR (AB=(parent OR parents OR parental OR mother OR father OR care*giver OR guardian* OR carer* OR paternal OR maternal))
2	(TI=(children OR adolescent* OR adolescence OR youth* OR child OR teenager* OR pediatric* OR paediatric* OR kid* OR teen* OR 'young person' OR 'young people' OR boy* OR girl* OR juvenile*)) OR (AB=(children OR adolescent* OR adolescence OR youth* OR child OR teenager* OR pediatric* OR paediatric* OR kid* OR teen* OR 'young person' OR 'young people' OR boy* OR girl* OR juvenile*))
3	(TI=('attention deficit disorder*' OR 'attention deficit hyperactive disorder*' OR adhd OR 'attention deficit disorder with hyperactivity' OR 'eating disorder*' OR 'eating problem*' OR 'feeding and eating disorders' OR 'emerging personality disorder*' OR 'emerging personality problem*' OR 'personality disorder' OR 'externalising disorder*' OR 'externalising problem*' OR 'externalizing disorder*' OR 'externalizing problem*' OR 'oppositional defiant disorder*' OR 'oppositional defiant problem*' OR 'attention deficit and disruptive behavior disorders' OR psychos* OR 'psychotic disorder*' OR 'psychotic problem*' OR anxiety OR depression OR depressive OR 'obsessive compulsive disorder' OR 'ocd' OR phobia OR phobic OR 'mood disorder' OR 'anxiety disorder' OR 'panic disorder' OR agoraphobia OR 'internalising problem*' OR 'internalising disorder*' OR 'internalizing problem*' OR 'internalizing disorder*)) OR (AB=('attention deficit disorder*' OR 'attention deficit hyperactive disorder*' OR adhd OR 'attention deficit disorder with hyperactivity' OR 'eating disorder*' OR 'eating problem*' OR 'feeding and eating disorders' OR 'emerging personality disorder*' OR 'emerging personality problem*' OR 'personality disorder' OR 'externalising disorder*' OR 'externalising problem*' OR 'externalizing disorder*' OR 'externalizing problem*' OR 'oppositional defiant disorder*' OR 'oppositional defiant problem*' OR 'attention deficit and disruptive behavior disorders' OR psychos* OR 'psychotic disorder*' OR 'psychotic problem*' OR anxiety OR depression OR depressive OR 'obsessive compulsive disorder' OR 'ocd' OR phobia OR phobic OR 'mood disorder' OR 'anxiety disorder' OR 'panic disorder' OR agoraphobia OR 'internalising problem*' OR 'internalising disorder*' OR 'internalizing problem*' OR 'internalizing disorder*))
4	(TI=(intervention OR treatment OR therapy OR 'peer support' OR 'support group' OR trial OR psychotherapy OR 'group support' OR 'talking therapy' OR counselling OR 'group therapy' OR 'group intervention' OR 'trauma informed' OR 'self help group' OR 'peer group')) OR (AB=(intervention OR treatment OR therapy OR 'peer support' OR 'support group' OR trial OR psychotherapy OR 'group support' OR 'talking therapy' OR

	counselling OR 'group therapy' OR 'group intervention' OR 'trauma informed' OR 'self help group' OR 'peer group'))
5	(TI=(stress OR anxiety OR depression OR burnout OR worry* OR 'parent* satisfaction*' OR wellbeing OR 'mental health')) OR (AB=(stress OR anxiety OR depression OR burnout OR worry* OR 'parent* satisfaction*' OR wellbeing OR 'mental health'))
6	#5 AND #4 AND #3 AND #2 AND #1
7	(TI=(anxiety OR depression OR depressive OR 'obsessive compulsive disorder' OR 'ocd' OR phobia OR phobic OR 'mood disorder' OR 'anxiety disorder' OR 'panic disorder' OR agoraphobia OR 'internalising problem*' OR 'internalising disorder*' OR 'internalizing problem*' OR 'internalizing disorder*)) OR (AB=(anxiety OR depression OR depressive OR 'obsessive compulsive disorder' OR 'ocd' OR phobia OR phobic OR 'mood disorder' OR 'anxiety disorder' OR 'panic disorder' OR agoraphobia OR 'internalising problem*' OR 'internalising disorder*' OR 'internalizing problem*' OR 'internalizing disorder*))
8	#6 NOT #7

Database: EMBASE

Search	Terms
1	'parent'/exp OR parent OR 'parents'/exp OR parents OR parental OR 'mother'/exp OR mother OR 'father'/exp OR father OR care*giver OR guardian* OR carer* OR paternal OR 'maternal'/exp OR maternal:ti,ab
2	children OR adolescent* OR adolescence OR youth* OR child OR teenager* OR pediatric* OR paediatric* OR kid* OR teen* OR 'young person' OR 'young people' OR boy* OR girl* OR juvenile*:ti,ab
3	'attention deficit disorder*' OR 'attention deficit hyperactive disorder*' OR adhd OR 'attention deficit disorder with hyperactivity' OR 'eating disorder*' OR 'eating problem*' OR 'feeding and eating disorders' OR 'emerging personality disorder*' OR 'emerging personality problem*' OR 'personality disorder' OR 'externalising disorder*' OR 'externalising problem*' OR 'externalizing disorder*' OR 'externalizing problem*' OR 'oppositional defiant disorder*' OR 'oppositional defiant problem*' OR 'attention deficit and disruptive behavior disorders' OR psychos* OR 'psychotic disorder*' OR 'psychotic problem*' OR anxiety OR depression OR depressive OR 'obsessive compulsive disorder' OR 'ocd' OR phobia OR phobic OR 'mood disorder' OR 'anxiety disorder' OR 'panic disorder' OR agoraphobia OR 'internalising problem*' OR 'internalising disorder*' OR 'internalizing problem*' OR 'internalizing disorder*':ti,ab
4	intervention OR treatment OR therapy OR 'peer support' OR 'support group' OR trial OR psychotherapy OR 'group support' OR 'talking therapy' OR counselling OR 'group therapy' OR 'group intervention' OR 'trauma informed' OR 'self help group' OR 'peer group':ti,ab
5	stress OR anxiety OR depression OR burnout OR worry* OR 'parent* satisfaction*' OR wellbeing OR 'mental health':ti,ab
6	#1 AND #2 AND #3 AND #4 AND #5
7	anxiety OR depression OR depressive OR 'obsessive compulsive disorder' OR 'ocd' OR phobia OR phobic OR 'mood disorder' OR 'anxiety disorder':ab,ti OR 'panic disorder' OR agoraphobia OR 'internalising problem*' OR 'internalising disorder*' OR 'internalizing problem*' OR 'internalizing disorder*':ab,ti
8	#6 NOT #7
