# Supplementary material for: Interventions to support parents and carers of young people with mental health difficulties: a systematic review protocol
Source: BMJ Open. 2023 Jun 9;13(6):e073940. doi: 10.1136/bmjopen-2023-073940 (PMC10277097; doi:10.1136/bmjopen-2023-073940)
Supplement: Author's manuscript [file bmjopen-2023-073940.draft_revisions.pdf]

BMJ Open is committed to open peer review. As part of this commitment we make the peer review history of every article we publish publicly available.

When an article is published we post the peer reviewers' comments and the authors' responses online. We also post the versions of the paper that were used during peer review. These are the versions that the peer review comments apply to.

The versions of the paper that follow are the versions that were submitted during the peer review process. They are not the versions of record or the final published versions. They should not be cited or distributed as the published version of this manuscript.

BMJ Open is an open access journal and the full, final, typeset and author-corrected version of record of the manuscript is available on our site with no access controls, subscription charges or pay-per-view fees (http://bmjopen.bmj.com).

If you have any questions on BMJ Open's open peer review process please email info.bmjopen@bmj.com

BMJ Open

A systematic review protocol of interventions to support parents and carers of young people with mental health difficulties

Journal:	*BMJ Open*
Manuscript ID	bmjopen-2023-073940
Article Type:	Protocol
Date Submitted by the Author:	22-Mar-2023
Complete List of Authors:	Martin, Faith; Cardiff University<br>Dahmash, Dania; Coventry University<br>Tsang, Anthony; King's College London<br>Glover, Sarah; Parental Minds Community Interest Company<br>Duncan, Charlie; British Association for Counselling and Psychotherapy<br>Halligan, Sarah; University of Bath, Department of Psychology
Keywords:	MENTAL HEALTH, Systematic Review, PUBLIC HEALTH, Adolescent

SCHOLARONE™
Manuscripts

A systematic review protocol of interventions to support parents and carers of young people with mental health difficulties

AUTHORS

Dr Faith Martin. School of Psychology, Cardiff University, 70 Park Pl, Cardiff CF10 3AS. Email martinf8@cardiff.ac.uk

Dr Dania Dahmash. Coventry University, Centre for Intelligent Healthcare, Coventry University, Richard Crossman Building, Jordan Well, Coventry CV1 5RW, UK. Email: ad9819@coventry.ac.uk

Anthony Tsang. King's Technology Evaluation Centre, London Institute for Healthcare Engineering, King's College London. 1 Lambeth Palace Rd, South Bank, London, SE1 7EU, UK. Email: anthony.t@live.co.uk.

Mrs Sarah Glover. Parental Minds Community Interest Company, UK. Email: parentplus@parentalminds.org.uk

Charlie Duncan. Research Department, BACP House, 15 St John's Business Park, Lutterworth, LE17 4HB Email charlie.duncan@bacp.co.uk

Professor Sarah Halligan. University of Bath, Department of Psychology, University of Bath, Claverton Down Campus, Bath BA2 7AY, UK. Email: slh54@bath.ac.uk

CORRESPONDING AUTHOR:

Dr Dania Dahmash. Coventry University, Centre for Intelligent Healthcare, Coventry University, Richard Crossman Building, Jordan Well, Coventry CV1 5RW, UK. Email ad9818@coventry.ac.uk

For peer review only

Abstract

Introduction: Globally 8-14% of children and young people (CYP) have a diagnosable mental health condition, many of whom receive no formal interventions. Parents/carers of CYP experience stress and distress owing to the mental health difficulties encountered by their CYP due to the lack of resources and support. Currently, little is known about 1) the content of interventions developed to support parents/carers nor 2) how effective interventions are at improving parents'/carers' wellbeing. The planned review aims to address these two gaps.

Method and analysis: A systematic review will be conducted to identify any study that describes an intervention aiming at least in part to support parents/carers with the impact of CYP (5-18 years) mental health difficulties, and to review any randomised-controlled trials (RCTs) of these interventions. The following databases will be searched: MEDLINE; PsycINFO; CINAHL; AMED; EMBASE; Web of Science Core Collection and Cochrane Library CENTRAL, without and limitations applied. Analysis of the content of interventions will be structured using the Template for Intervention Description and Replication checklist as a framework. The effect of any RCTs on parents'/carers' outcomes (including wellbeing, satisfaction with parenting, mental health) will be extracted and assessed using the Cochrane Risk-of-Bias Tool. Data will be synthesised narratively, with meta-analysis of RCT results, if appropriate.

Ethical consideration and dissemination: The protocol has been registered on PROSPERO under the registration number: CRD42022344453 and approved by Coventry University Ethical Committee (reference number: P139611). Results will be shared in academic publications and in accessible formats using social media and public webinars.

Keywords:

For peer review only

Children; young people; mental health; parent; carers; interventions; parental needs; systematic review

Strengths and limitations of this study

1- First systematic review examining interventions aiming to support parents' and carers' of children and young people with mental health difficulties
2- The systematic review and meta-analysis, if possible, of randomised controlled trials will allow clarification of the effectiveness of these interventions.
3- Inclusion of the narrative analysis of the intervention components is a major strength, providing insight into the details of what the interventions reviewed cover.
4- We will use a rigorous method and the results of the review will be reported in accordance with Preferred Reporting Items for Systematic Review and Meta-Analysis reporting guidelines
5- The review is restricted to studies that are published in English only

For peer review only

INTRODUCTION

Mental health conditions amongst children and young people (CYP) are increasingly common: globally around 8% of 5-9 year olds and 14% of 10-18 years old have a diagnosable mental health condition (1). In the UK, approximately one in six school-aged children have a mental health problem (2). Demand is outstripping service provision, with lengthy waiting times and only around a quarter (27%) of CYP who need mental health support in the UK are receiving it (3). This may place greater pressure on families to manage the difficulties on their own. Therefore, parents and carers (hereafter "parents") are often experiencing stress and distress in relation to their CYP's mental health difficulty, including depression, managing stigma, anxiety, and work and financial pressures (4-6).

Policy recommendations include seeing parents as partners in their CYP's care (7), however little attention is paid to the parents' own needs. The World Health Organization's (WHO) report describes the importance of supporting parent mental health, however focuses on parents with pre-existing mental health conditions and new parents (1). There is reference to the needs of parents of CYP with developmental delays or difficulties, highlighting that they are likely to experience high levels of distress. Whilst clearly vital, there is a need to also support parents with the understandable distress of having a CYP with a mental health difficulty.

Interventions have been developed that include parents to address CYP mental health. Parent training, family therapy, and parent-co-delivered/led cognitive behavioural therapy all, to some extent, involve the parent (8-10). However, the focus is frequently not on the parents' wellbeing, nor on addressing the impact of CYP mental health difficulties on the parent. The content of interventions (or elements of interventions) that specifically aim to support parents has not yet been identified or summarised. This is important, as identification of the content can support evaluation of effectiveness, for example interventions may be focusing on just one

For peer review only

elements of parental distress by providing information, and thus not necessarily achieving good outcomes. The content of the interventions can be compared to the needs that have been identified for parents/carers(11).

Given the lack of policy guidelines and existing evidence synthesis, this systematic review protocol outlines the process for identifying literature which addresses the following research questions:

1- What is the content of interventions or intervention components that have been designed to address parents' wellbeing in the context of CYP mental health difficulties
2- How effective are these interventions in terms of improving parents' wellbeing outcomes, family wellbeing and mental health service utilisation?

METHOD AND ANALYSIS

The study has been registered in the International Prospective Register of Systematic Reviews (PROSPERO: CRD42022344453). This protocol follows the Preferred Reporting Items for Systematic Review and Meta-Analyses for Protocols (PRISMA-P) 2015 guidelines (12). The completed systematic review will adhere to the reporting guidelines of PRISMA-2020 (13).

Study selection criteria

Participants

The participants to be included in this review will be parents of CYP (aged 5-18) who have a diagnosis of a mental health condition. This age range reflects the upper limit of most CYP specific mental health services and covers CYP before they have gone to university, therefore are more likely to still be at home with parents, with the lower limit relating to youngest age of onset for most conditions (14). To be included, the majority (>50%) of the CYP must be within

For peer review only

that age range. The CYP must have at least one diagnosed mental health condition from depression, anxiety disorders, psychoses, oppositional defiant and other externalising disorders, labels of emerging personality disorders, eating disorders, and attention deficit (hyperactive) disorders. "Parents" covers all adults (aged 18 years and over) in a formal parenting role, including biological parents, step-parents, other relatives assuming a parenting role, non-biological and adoptive parents, foster-carers, and other adults in legal guardian roles. The review does not cover parents/carers of CYP with special educational needs, including autism spectrum, as there are existing reviews and services and needs are different (15). Where the focus is on CYP with mental health difficulties and within that include less than 50% CYP with developmental/special educational needs, studies would be included. Study samples can be drawn from any setting, for example, supporting parents of CYP on waiting-lists, or receiving care through voluntary or statutory services, providing that the CYP has a diagnosed condition.

Intervention

The intervention must include a component specifically designed to meet the parents'/carers' needs – be that information, emotional or social support. Interventions that train parents/carers to provide therapy or behavioural management techniques, where the focus of the intervention is solely on changing CYP outcome, will not be included. Interventions that include therapy for the child but also support for parents/carers will be included. However, for research question two, the intervention need not be described in full in the study however must provide sufficient evidence (e.g. brief description, reference to a study that does describe the intervention) of relevance to the review.

Comparison

For peer review only

This only applies for research question two. The comparison can be with any control group, including treatment as usual or active controls.

Outcomes

For research question two, outcomes must be measured using validated tools only. Relevant outcomes are parental mental health (e.g., depression, anxiety, stress); parent-CYP relationships; satisfaction with parenting; parenting self-efficacy; family wellbeing; or parent/carer health service utilisation. Search terms relating to the outcomes are used to identify studies for research question one also, to identify interventions that address our outcomes of interest.

Study design

For research question one, relating to the content of interventions, studies must provide a description of an intervention with at least one component to support parents'/carers' needs or wellbeing. Studies can be of any design, and do not need to report any intervention outcomes. For research question two, randomised controlled trials (RCTs) will be included and assessed following Cochrane Reviewing guidelines (16). We will also include a narrative synthesis of studies that describe an intervention and its early stage testing (e.g. feasibility trials, case series).

Search strategy

The following bibliographic databases were searched: MEDLINE(EBSCO); PsycINFO (Ovid); CINAHL Ultimate (EBSCO); AMED(EBSCO); EMBASE(Ovid); Web of Science Core Collection; Cochrane Library databases including: Cochrane Database of Systematic Reviews, Cochrane Central Register of Controlled Trials, Database of Abstracts of Reviews of

For peer review only

Effects, Health Technology Assessment Database, and NHS Economic Evaluation Database. No filters were applied on the search.

The reference lists of the any identified systematic reviews will be screened for additional studies not captured by the database searching. Supplementary searches will be performed in the form of forward and backward citation using Citationchaser to potentially identify additional studies(17).

The search terms will include five blocks of terms, all combined with "AND". These terms cover:

1. Parents or carers
2. Children and young people
3. Mental health problems
4. Intervention or treatment or therapy or peer support psychotherapy or counselling or group therapy.
5. Outcomes of interest including stress, anxiety, depression, burnout, or parenting satisfaction

A scoping search was conducted using the above listed terms, in which (3,827,850) records were identified. This scoping search was used to refine search terms, particularly use of subject headings. To manage the downloading of records and the workload thereafter, two complementary search strategies will be used (supplementary file 1 and 2). The first search strategy will focus on studies relating to CYP anxiety and/or depression. This is owing to these conditions being very common and representing a large portion of the overall records from the scoping search. The second search strategy then will identify studies linked to all other included CYP mental health difficulties. All criteria for inclusion/exclusion and data extraction will be conducted in the same way – this split is merely to manage the search results rather than conduct separate reviews. (If search 1 identifies studies that extend beyond anxiety and or depression to other included conditions, they will be included during screening).

For peer review only

The planned analysis includes considering results in relation to CYP difficulty, as well as examining similarities/differences in intervention content and efficacy across CYP difficulties.

Patient and Public involvement

The scope of the review has been defined through discussion with parents of CYP with mental health difficulties (including with co-author SG's group "Parental Minds"). This PPI engagement helped to design the study, refine the scope, and specify the protocol, including the exclusion of autistic spectrum disorders and development difficulties from this review.

During the review, we will consult with a group of parents/carers as needed on decisions made, for example the relevance of interventions to parents' needs and thus inclusion in the review. Initial synthesis and interpretation of findings will be shared with our PPI group for discussion and refinement. PPI involvement, particularly in writing interpretation and recommendations, is planned for the writing of all outputs, design of non-academic papers, and wider dissemination activities.

DATA COLLECTION AND ANALYSIS

Selection process

Rayyan software will be used to manage records (18, 19). This facilitates blind decisions for inclusion/exclusion. Inclusion/exclusion criteria will be reviewed by stakeholders. A two-step process will be used. First, all titles and abstracts will be screened against the inclusion/exclusion criteria by two reviewers independently. Second, reports will be obtained for short-listed records and inclusion/exclusion will be conducted by two reviewers independently. Any discrepancies at either step will be resolved by a third reviewer if needed.

Data extraction and quality appraisal

For peer review only

A data extraction form will be created and piloted by at least two reviewers. Data will be extracted by two reviewers (D.D and A.T) and all data will be checked by a third reviewer. Data will be extracted into an Excel sheet. Discrepancies will be resolved by discussion with a third reviewer. For both question extracted data will include, study characteristics (author, study design, setting , aim of the study and sample size) and population characteristics (CYP age, Parents/carers age, ethnicity and relationship to CYP).

For research question 1, detailed information about the types of interventions developed will be extracted independently by two reviewers using the Template for Intervention Description and Replication (TIDieR) checklist as basis to assess the completeness of the descriptions, therefore in relation to this research question, this also covers the quality appraisal(20). This checklist includes: 1) descriptions of needs addressed, 2) aims of the intervention, 3) intervention content, 4) materials used, 5) inclusion criteria, 6) number and duration of sessions, 7) who delivers content and how, 8) where/what modality, 9) personalisation or tailoring of the intervention, 10) underpinning theory, or approach. Information about implementation, including structure and content of training and supervision, and resource requirements will be included. If information is not specifically provided in a study, we will consult protocol and intervention design reports, and contact authors for further information as necessary. Where studies describe interventions for parents/carers and children, data will be extracted relating to the intervention or components for parents/carers. Data will also be extracted relating to any proposed mechanisms of how the intervention may operate.

For research question two, details of the participant characteristics, intervention itself (covered in research question one), and comparison group(s), and details of outcomes (e.g. validated measures of parent/carer mental health (depression, anxiety, stress); parent-CYP relationships; satisfaction with parenting; family wellbeing; and parent/carer health service utilisation) will be extracted. CYP MH service utilisation will also be extracted, if reported in parent/carer only

For peer review only

interventions, to elucidate any impact on this important service outcome. Comparisons between intervention and control follow-up data points will be extracted, with effect sizes, to indicate the impact of the intervention in comparison to control. Quality will be appraised using the Cochrane Risk of Bias Tool(Version 2) (16).

Data synthesis

For research question one, the aim is to describe in detail and categorise approaches to interventions to support parents/carers, mapping these onto parent/carer needs and describing underpinning theoretical approaches. No established a priori framework to categorise these interventions exists. Content analysis will be used to synthesise the data (21-23), within each data field from the TIDieR checklist. For example, codes for the underpinning theories will be created. Two reviewers' will independently analyse and code the data, with discussion of disagreements taken to a third reviewer, parent/carer working group, or wider stakeholder group if required. Content analysis of intervention details will be tabulated, capturing frequency of inclusion of different intervention content and other intervention details. Where multiple studies describe the same intervention, extracted data will be synthesised into a single entry for that intervention. Findings will also be grouped by CYP diagnosis and compared across different diagnoses.

For research question two, Cochrane systematic review of interventions methods will be followed (16). Mean differences will be used for continuous measures and for outcomes where different measures are used (e.g., anxiety, depression), standardised mean differences. In the absence of significant heterogeneity and if there are sufficient studies, meta-analysis will be considered using random effects models using RevMan 5(24). This will be conducted where possible in relation to each outcome variable group (e.g. parents' depression, parents' stress). In the presence of significant heterogeneity a narrative synthesis approach will be followed

For peer review only

using Cochrane guidance (25). If there are sufficient studies, stratified analyses will be conducted by CYP diagnoses, intervention type, and active versus inactive comparators. Sensitivity analyses will be performed excluding studies at high risk of bias. Publication bias will be explored using funnel plots if there are sufficient studies. Identified as important in PPI discussions, one reviewer will review the summaries, and comment on the extent to which the specific identified outcome measures are patient-centered. A vital element of the analysis will be to compare and contrast the outcomes for interventions in relation to different CYP mental health difficulties. Interventions that do not select participants based on CYP mental health difficulty will be compared to those for specific problems, as well as comparisons between specific CYP mental health difficulties. If possible, these sub-group analyses will comprise of meta-analyses of studies by CYP mental health difficulty, however if meta-analyses are not possible (for example, if high levels of heterogeneity as measured by I^2), narrative synthesis will consider the potential relevance of CYP mental health difficulty type to on reported outcomes, and considering any reported cross-cutting factors such a risk to CYP and duration of CYP mental health difficulty. Sub-group analysis will also be considered to examine differences in relation to whether CYP is receiving or awaiting care; whether care if via statutory or non-statutory services; and by intervention type if there are clear differences in theoretical approaches to the interventions.

ETHICS AND DISSEMINATION

This study was approved by Coventry University ethical committee, reference number: P139611.

Dissemination will use multiple channels such as YouTube and social media, in addition to traditional academic outputs. We will disseminate findings via all our collaborators and

For peer review only

partners in the NHS and charitable sectors to use their networks to share the findings widely. We particularly seek to reach UK policy makers and clinicians using forums such the Future NHS Collaboration platform. Parents/carers involved in the project will be sent the final outputs via email links/hard copy.

COMPETING INTERESTS

All authors have completed the ICMJE uniform disclosure form. SG is an author and the director of Parental Minds, which provide services to parent of children with mental health. The authors have no competing interests to declare.

AUTHORS' CONTRIBUTIONS

FM created the idea and led the project, conduced the databases searches and contributing to all stages. FM and DD prepared the manuscript draft. SG, CD, AT and SH contributed to the writing and the reviewing of and the manuscript. Study selection and data collection will be conducted by FM, DD and AT. FM, DD, SG, CD, AT will be involved in the synthesis and interpretation of the data. FM, DD, SG, CD, AT, and SH along with the parent/carer panel will be involved in the final drafting and dissemination of findings. FM, DD, SG, CD, AT, and SH read and approved the final manuscript.

FUNDING

This work was supported by the National Institute of Health Research (NIHR); grant number: NIHR20302.

For peer review only

ACKNOWLEDGEMENTS

We would like to acknowledge and thank the members of the PPI group who have provided invaluable input to date.

LIST OF ABBREVIATIONS

CAMHS – Child and Adolescent Mental Health Service

MH-Mental health

CYP- Child and Young Person

TIDieR- Template for intervention description and replication

For peer review only

REFERENCES

1. WHO. World mental health report: transforming mental health for all. World Health Organization (WHO), accessed January 2023 from https://www.who.int/publications/i/item/9789240049338; 2022.
2. Newlove-Delgado T, Williams T, Robertson K, McManus S, Sadler K, Vizard T, et al. Mental Health of Children and Young People in England 2021-wave 2 follow up to the 2017 survey. 2021.
3. Grimm F, Alcock B, Butler J, Fernandez Crespo R, Davies A, Peytrignet S, et al. Improving children and young people's mental health services. 2022.
4. Shenaar-Golan V, Wald N, Yatzkar U. Parenting a Child with Mental Health Problems: the Role of Self-Compassion. Mindfulness. 2021;12(11):2810-9.
5. Whitlock J, Lloyd-Richardson E, Fisseha F, Bates T. Parental Secondary Stress: The Often Hidden Consequences of Nonsuicidal Self-Injury in Youth. Journal of clinical psychology. 2018;74(1):178-96.
6. Gerkensmeyer JE, Perkins SM, Day J, Austin JK, Scott EL, Wu J. Maternal depressive symptoms when caring for a child with mental health problems. Journal of Child and Family Studies. 2011;20(5):685-95.
7. Care Quality Commission. Review of children and young people's mental health services. Care Quality Commission, London. 2017.
8. Reyno SM, McGrath PJ. Predictors of parent training efficacy for child externalizing behavior problems – a meta-analytic review. Journal of Child Psychology and Psychiatry. 2006;47(1):99-111.
9. Jewell T, Blessitt E, Stewart C, Simic M, Eisler I. Family therapy for child and adolescent eating disorders: a critical review. Family Process. 2016;55(3):577-94.
10. Lawrence P, Parkinson M, Jasper B, Creswell C, Halligan S. Supporting parents of children and young people with anxiety and depressive disorders, an opportunity not to be missed: A scoping review. Lancet Psychiatry. 2020;in press.

11. Armitage S, Parkinson M, Halligan S, Reynolds SJJoC, Studies F. Mothers' experiences of having an adolescent child with depression: An interpretative phenomenological analysis. 2020;29:1617-29.

12. Moher D, Shamseer L, Clarke M, Ghersi D, Liberati A, Petticrew M, et al. Preferred reporting items for systematic review and meta-analysis protocols (PRISMA-P) 2015 statement. 2015;4(1):1-9.

13. Page MJ, McKenzie JE, Bossuyt PM, Boutron I, Hoffmann TC, Mulrow CD, et al. The PRISMA 2020 statement: an updated guideline for reporting systematic reviews. 2021;88:105906.

14. Kessler RC, Berglund P, Demler O, Jin R, Merikangas KR, Walters EE. Lifetime Prevalence and Age-of-Onset Distributions of DSM-IV Disorders in the National Comorbidity Survey Replication. Archives of General Psychiatry. 2005;62(6):593-602.

15. Frantz R, Hansen SG, Machalicek W. Interventions to promote well-being in parents of children with autism: A systematic review. Review Journal of Autism and Developmental Disorders. 2018;5:58-77.

16. Higgins JP, Thomas J, Chandler J, Cumpston M, Li T, Page MJ, et al. Cochrane handbook for systematic reviews of interventions: John Wiley & Sons; 2019.

17. Haddaway N, Grainger M, Gray CJZ, Feb. citationchaser: An R package and Shiny app for forward and backward citations chasing in academic searching. 2021;16.

18. Ouzzani M, Hammady H, Fedorowicz Z, Elmagarmid AJSr. Rayyan—a web and mobile app for systematic reviews. 2016;5:1-10.

19. Covidence. Covidence systematic review software, Veritas Health Innovation.

20. Hoffmann TC, Glasziou PP, Boutron I, Milne R, Perera R, Moher D, et al. Better reporting of interventions: template for intervention description and replication (TIDieR) checklist and guide. 2014;348.

21. Ash T, Agaronov A, Young TL, Aftosmes-Tobio A, Davison KKJIJoBN, Activity P. Family-based childhood obesity prevention interventions: a systematic review and quantitative content analysis. 2017;14(1):1-12.

For peer review only

22. Marston N, Stavnes K, Van Loon LM, Drost LM, Maybery D, Mosek A, et al. A content analysis of Intervention Key Elements and Assessments (IKEA): What's in the black box in the interventions directed to families where a parent has a mental illness? 2016;37(2):112-28.

23. Hsieh H-F, Shannon SEJQhr. Three approaches to qualitative content analysis. 2005;15(9):1277-88.

24. Manager R. Version 5.4, The Cochrane Collaboration. The Nordic Cochrane Centre Copenhagen, Denmark; 2020.

25. Campbell M, McKenzie JE, Sowden A, Katikireddi SV, Brennan SE, Ellis S, et al. Synthesis without meta-analysis (SWiM) in systematic reviews: reporting guideline. 2020;368.

For peer review only

Search terms used for depression and anxiety studies:

Database: Medline

Search	Term
S1 (Parent)	TI ((parent or parents or parental or mother or father or care*giver or guardian* or carer* or paternal or maternal)) OR AB ((parent or parents or parental or mother or ather or care*giver or guardian* or carer* or paternal or maternal)) OR MM ("Parents+")
S2 (children)	TI ((children or adolescent* or adolescence or youth* or child or teenager* or pediatric* or paediatric* or kid* or teen* or young person or young people or boy* or girl* or juvenile*)) OR AB ((children or adolescent* or adolescence or youth* or child or teenager* or pediatric* or paediatric* or kid* or teen* or young person or young people or boy* or girl* or juvenile*)) OR MH ("Child+") OR MM ("Adolescent")
S3 (Mental Health)	TI ((Anxiety or depression or depressive or "obsessive compulsive disorder" or "OCD" or phobia or phobic or mood disorder or anxiety disorder or panic disorder or agoraphobia or internalising problem* or internalising problem* or internalizing problem* or internalizing disorder*)) OR AB ((Anxiety or depression or depressive or "obsessive compulsive disorder" or "OCD" or phobia or phobic or mood disorder or anxiety disorder or panic disorder or agoraphobia or internalising problem* or internalising problem* or internalizing problem* or internalizing disorder*) OR (MH "Depressive Disorder") OR (MH "Depressive Disorder, Major") OR (MH "Depressive Disorder, Treatment-Resistant") OR (MH "Dysthymic Disorder") OR (MM "Anxiety Disorders+"))
S4 (Intervention)	TI (intervention or treatment or therapy or peer support or support group or trial or psychotherapy or group support or talking therapy or counselling or group therapy or group intervention or trauma informed) OR AB (intervention or treatment or therapy or peer support or support group or trial or psychotherapy or group support or talking therapy or counselling or group therapy or group intervention or trauma informed) OR (MH "Psychotherapy") OR (MH "Self-Help Groups") OR (MH "Peer Group")
S5 (outcome)	TX (stress or anxiety or depression or burnout or worry* or "parent* satisfaction*" or wellbeing or mental health)
S6	S1 AND S2 AND S3 AND S4 AND S5

Database: PsycINFO

Search	Term
S1 (Parent)	TI ((parent or parents or parental or mother or father or care*giver or guardian* or carer* or paternal or maternal)) OR AB ((parent or parents or parental or mother or ather or care*giver or guardian* or carer* or paternal or maternal)) OR MM ("Parents+")
S2 (children)	TI ((children or adolescent* or adolescence or youth* or child or teenager* or pediatric* or paediatric* or kid* or teen* or young person or young people or boy* or girl* or juvenile*)) OR AB ((children or adolescent* or adolescence or youth* or child or teenager* or pediatric* or paediatric* or kid* or teen* or young person or young people or boy* or girl* or juvenile*)) OR MH ("Child+") OR MM ("Adolescent")
S3 (Mental Health)	TI ((Anxiety or depression or depressive or "obsessive compulsive disorder" or "OCD" or phobia or phobic or mood disorder or anxiety disorder or panic disorder or agoraphobia or internalising problem* or internalising problem* or internalizing problem* or internalizing disorder*)) OR AB ((Anxiety or depression or depressive or "obsessive compulsive disorder" or "OCD" or phobia or phobic or

For peer review only

	mood disorder or anxiety disorder or panic disorder or agoraphobia or internalising problem* or internalising problem* or internalizing problem* or internalizing disorder*) OR (MH "Depressive Disorder") OR (MH "Depressive Disorder, Major") OR (MH "Depressive Disorder, Treatment-Resistant") OR (MH "Dysthymic Disorder") OR (MM "Anxiety Disorders+"))
S4 (Intervention)	TI (intervention or treatment or therapy or peer support or support group or trial or psychotherapy or group support or talking therapy or counselling or group therapy or group intervention or trauma informed) OR AB (intervention or treatment or therapy or peer support or support group or trial or psychotherapy or group support or talking therapy or counselling or group therapy or group intervention or trauma informed) OR (MH "Psychotherapy") OR (MH "Self-Help Groups") OR (MH "Peer Group")
S5 (outcome)	TX (stress or anxiety or depression or burnout or worry* or "parent* satisfaction*" or wellbeing or mental health)
S6	S1 AND S2 AND S3 AND S4 AND S5

Database: CINAHL ULTIMATE

Search	Term
S1 (Parent)	TI ((parent or parents or parental or mother or father or care*giver or guardian* or carer* or paternal or maternal)) OR AB ((parent or parents or parental or mother or ather or care*giver or guardian* or carer* or paternal or maternal)) OR MM ("Parents+")
S2 (children)	TI ((children or adolescent* or adolescence or youth* or child or teenager* or pediatric* or paediatric* or kid* or teen* or young person or young people or boy* or girl* or juvenile*)) OR AB ((children or adolescent* or adolescence or youth* or child or teenager* or pediatric* or paediatric* or kid* or teen* or young person or young people or boy* or girl* or juvenile*)) OR MH ("Child+") OR MM ("Adolescent")
S3 (Mental Health)	TI ((Anxiety or depression or depressive or “obsessive compulsive disorder” or “OCD” or phobia or phobic or mood disorder or anxiety disorder or panic disorder or agoraphobia or internalising problem* or internalising problem* or internalizing problem* or internalizing disorder*)) OR AB ((Anxiety or depression or depressive or “obsessive compulsive disorder” or “OCD” or phobia or phobic or mood disorder or anxiety disorder or panic disorder or agoraphobia or internalising problem* or internalising problem* or internalizing problem* or internalizing disorder*) OR (MH "Depressive Disorder") OR (MH "Depressive Disorder, Major") OR (MH "Depressive Disorder, Treatment-Resistant") OR (MH "Dysthymic Disorder") OR (MM "Anxiety Disorders+"))
S4 (Intervention)	TI (intervention or treatment or therapy or peer support or support group or trial or psychotherapy or group support or talking therapy or counselling or group therapy or group intervention or trauma informed) OR AB (intervention or treatment or therapy or peer support or support group or trial or psychotherapy or group support or talking therapy or counselling or group therapy or group intervention or trauma informed) OR (MH "Psychotherapy") OR (MH "Self-Help Groups") OR (MH "Peer Group")
S5 (outcome)	TX (stress or anxiety or depression or burnout or worry* or "parent* satisfaction*" or wellbeing or mental health)
S6	S1 AND S2 AND S3 AND S4 AND S5

Database: AMED

Search	Term
S1 (Parent)	TI ((parent or parents or parental or mother or father or care*giver or guardian* or carer* or paternal or maternal)) OR AB ((parent or parents or parental or mother or ather or care*giver or guardian* or carer* or paternal or maternal)) OR MM ("Parents+")
S2 (children)	TI ((children or adolescent* or adolescence or youth* or child or teenager* or pediatric* or paediatric* or kid* or teen* or young person or young people or boy* or girl* or juvenile*)) OR AB ((children or adolescent* or adolescence or youth* or child or teenager* or pediatric* or paediatric* or kid* or teen* or young person or young people or boy* or girl* or juvenile*)) OR MH ("Child+") OR MM ("Adolescent")
S3 (Mental Health)	TI ((Anxiety or depression or depressive or "obsessive compulsive disorder" or "OCD" or phobia or phobic or mood disorder or anxiety disorder or panic disorder or agoraphobia or internalising problem* or internalising problem* or internalizing problem* or internalizing disorder*)) OR AB ((Anxiety or depression or depressive or "obsessive compulsive disorder" or "OCD" or phobia or phobic or mood disorder or anxiety disorder or panic disorder or agoraphobia or internalising problem* or internalising problem* or internalizing problem* or internalizing disorder*) OR (MH "Depressive Disorder") OR (MH "Depressive Disorder, Major") OR (MH "Depressive Disorder, Treatment-Resistant") OR (MH "Dysthymic Disorder") OR (MM "Anxiety Disorders+"))
S4 (Intervention)	TI (intervention or treatment or therapy or peer support or support group or trial or psychotherapy or group support or talking therapy or counselling or group therapy or group intervention or trauma informed) OR AB (intervention or treatment or therapy or peer support or support group or trial or psychotherapy or group support or talking therapy or counselling or group therapy or group intervention or trauma informed) OR (MH "Psychotherapy") OR (MH "Self-Help Groups") OR (MH "Peer Group")
S5 (outcome)	TX (stress or anxiety or depression or burnout or worry* or "parent* satisfaction*" or wellbeing or mental health)
S7	S1 AND S2 AND S3 AND S4 AND S5

Database: The Cochrane Library (including the Cochrane Database of Systematic Reviews, the Cochrane Central Register of Controlled Trials (CENTRAL), the Database of Abstracts of Reviews of Effects, the Health Technology Assessment Database, and the NHS Economic Evaluation Database)

Search	Term
S1 (Parent)	TI ((parent or parents or parental or mother or father or care*giver or guardian* or carer* or paternal or maternal)) OR AB ((parent or parents or parental or mother or ather or care*giver or guardian* or carer* or paternal or maternal)) OR MM ("Parents+")
S2 (children)	TI ((children or adolescent* or adolescence or youth* or child or teenager* or pediatric* or paediatric* or kid* or teen* or young person or young people or boy* or girl* or juvenile*)) OR AB ((children or adolescent* or adolescence or youth* or child or teenager* or pediatric* or paediatric* or kid* or teen* or young person or young people or boy* or girl* or juvenile*)) OR MH ("Child+") OR MM ("Adolescent"))
S3 (Mental Health)	TI ((Anxiety or depression or depressive or "obsessive compulsive disorder" or "OCD" or phobia or phobic or mood disorder or anxiety disorder or panic disorder or agoraphobia or internalising problem* or internalising problem* or internalizing problem* or internalizing disorder*)) OR AB ((Anxiety or depression or

For peer review only

	depressive or “obsessive compulsive disorder” or “OCD” or phobia or phobic or mood disorder or anxiety disorder or panic disorder or agoraphobia or internalising problem* or internalising problem* or internalizing problem* or internalizing disorder*) OR (MH "Depressive Disorder") OR (MH "Depressive Disorder, Major") OR (MH "Depressive Disorder, Treatment-Resistant") OR (MH "Dysthymic Disorder") OR (MM "Anxiety Disorders+"))
S4 (Intervention)	TI (intervention or treatment or therapy or peer support or support group or trial or psychotherapy or group support or talking therapy or counselling or group therapy or group intervention or trauma informed) OR AB (intervention or treatment or therapy or peer support or support group or trial or psychotherapy or group support or talking therapy or counselling or group therapy or group intervention or trauma informed) OR (MH "Psychotherapy") OR (MH "Self-Help Groups") OR (MH "Peer Group")
S5 (outcome)	TX (stress or anxiety or depression or burnout or worry* or "parent* satisfaction*" or wellbeing or mental health)
S7	S1 AND S2 AND S3 AND S4 AND S5

Database: Web of Science Core Collection

Search	Terms
1	(TI=(parent OR parents OR parental OR mother OR father OR care*giver OR guardian* OR carer* OR paternal OR maternal)) OR (AB=(parent OR parents OR parental OR mother OR father OR care*giver OR guardian* OR carer* OR paternal OR maternal))
2	(TI=(children OR adolescent* OR adolescence OR youth* OR child OR teenager* OR pediatric* OR paediatric* OR kid* OR teen* OR 'young person' OR 'young people' OR boy* OR girl* OR juvenile*)) OR (AB=(children OR adolescent* OR adolescence OR youth* OR child OR teenager* OR pediatric* OR paediatric* OR kid* OR teen* OR 'young person' OR 'young people' OR boy* OR girl* OR juvenile*))
3	TI ((Anxiety or depression or depressive or “obsessive compulsive disorder” or “OCD” or phobia or phobic or mood disorder or anxiety disorder or panic disorder or agoraphobia or internalising problem* or internalising problem* or internalizing problem* or internalizing disorder*)) OR AB ((Anxiety or depression or depressive or “obsessive compulsive disorder” or “OCD” or phobia or phobic or mood disorder or anxiety disorder or panic disorder or agoraphobia or internalising problem* or internalising problem* or internalizing problem* or internalizing disorder*) OR (MH "Depressive Disorder") OR (MH "Depressive Disorder, Major") OR (MH "Depressive Disorder, Treatment-Resistant") OR (MH "Dysthymic Disorder") OR (MM "Anxiety Disorders+"))
4	(TI=(intervention OR treatment OR therapy OR 'peer support' OR 'support group' OR trial OR psychotherapy OR 'group support' OR 'talking therapy' OR counselling OR 'group therapy' OR 'group intervention' OR 'trauma informed' OR 'self help group' OR 'peer group')) OR (AB=(intervention OR treatment OR therapy OR 'peer support' OR 'support group' OR trial OR psychotherapy OR 'group support' OR 'talking therapy' OR counselling OR 'group therapy' OR 'group intervention' OR 'trauma informed' OR 'self help group' OR 'peer group'))
5	(TI=(stress OR anxiety OR depression OR burnout OR worry* OR 'parent* satisfaction*' OR wellbeing OR 'mental health')) OR (AB=(stress OR anxiety OR depression OR burnout OR worry* OR 'parent* satisfaction*' OR wellbeing OR 'mental health'))
6	#5 AND #4 AND #3 AND #2 AND #1

Database: EMBASE

Search	Terms
1	'parent'/exp OR parent OR 'parents'/exp OR parents OR parental OR 'mother'/exp OR mother OR 'father'/exp OR father OR care*giver OR guardian* OR carer* OR paternal OR 'maternal'/exp OR maternal:ti,ab
2	children OR adolescent* OR adolescence OR youth* OR child OR teenager* OR pediatric* OR paediatric* OR kid* OR teen* OR 'young person' OR 'young people' OR boy* OR girl* OR juvenile*:ti,ab
3	anxiety OR depression OR depressive OR 'obsessive compulsive disorder' OR 'ocd' OR phobia OR phobic OR 'mood disorder' OR 'anxiety disorder':ab,ti OR 'panic disorder' OR agoraphobia OR 'internalising problem*' OR 'internalising disorder*' OR 'internalizing problem*' OR 'internalizing disorder*':ab,ti
4	intervention OR treatment OR therapy OR 'peer support' OR 'support group' OR trial OR psychotherapy OR 'group support' OR 'talking therapy' OR counselling OR 'group therapy' OR 'group intervention' OR 'trauma informed' OR 'self help group' OR 'peer group':ti,ab
5	stress OR anxiety OR depression OR burnout OR worry* OR 'parent* satisfaction*' OR wellbeing OR 'mental health':ti,ab
6	#1 AND #2 AND #3 AND #4 AND #5

For peer review only

Search terms used excluding depression and anxiety terms:

Database: Medline

Search	Term
S1	TI ((parent or parents or parental or mother or father or care*giver or guardian* or carer* or paternal or maternal)) OR AB ((parent or parents or parental or mother or ather or care*giver or guardian* or carer* or paternal or maternal)) OR MM ("Parents+")
S2	TI ((children or adolescent* or adolescence or youth* or child or teenager* or pediatric* or paediatric* or kid* or teen* or young person or young people or boy* or girl* or juvenile*)) OR AB ((children or adolescent* or adolescence or youth* or child or teenager* or pediatric* or paediatric* or kid* or teen* or young person or young people or boy* or girl* or juvenile*)) OR MH ("Child+") OR MM ("Adolescent")
S3	TI ((attention deficit disorder* or “attention deficit hyperactive disorder* “or “ADHD”) OR AB (attention deficit disorder* or “attention deficit hyperactive disorder* “or “ADHD”)OR MH (“Attention Deficit Disorder with Hyperactivity”) OR (TI ((Eating disorder* or eating problem*)) OR AB ((Eating disorder* or eating problem*) OR MH(“Feeding and Eating Disorders+”) OR (TI ((Emerging personality disorder* or emerging personality problem*)) OR AB ((Emerging personality disorder* or emerging personality problem*)) OR MH (“Personality Disorder+”)) OR (TI ((Externalising disorder* or externalising problem* or externalizing disorder* or externalizing problem*) OR AB (Externalising disorder* or externalising problem* or externalizing disorder* or externalizing problem*) OR TX (“Externalising disorder”)) OR (TI ((Oppositional defiant disorder* or oppositional defiant problem*)) OR AB ((Oppositional defiant disorder* or oppositional defiant problem*) OR (MH“Attention Deficit and Disruptive Behavior Disorders+”)) OR (TI ((Psychos* or psychotic disorder* or psychotic problem*)) OR AB ((Psychos* or psychotic disorder* or psychotic problem*)) OR MH (“Psychotic Disorders+)) OR (TI ((Anxiety or depression or depressive or “obsessive compulsive disorder” or “OCD” or phobia or phobic or mood disorder or anxiety disorder or panic disorder or agoraphobia or internalising problem* or internalising problem* or internalizing problem* or internalizing disorder*)) OR AB ((Anxiety or depression or depressive or “obsessive compulsive disorder” or “OCD” or phobia or phobic or mood disorder or anxiety disorder or panic disorder or agoraphobia or internalising problem* or internalising problem* or internalizing problem* or internalizing disorder*) OR (MH "Depressive Disorder") OR (MH "Depressive Disorder, Major") OR (MH "Depressive Disorder, Treatment-Resistant") OR (MH "Dysthymic Disorder") OR (MM "Anxiety Disorders+"))
S4	TI (intervention or treatment or therapy or peer support or support group or trial or psychotherapy or group support or talking therapy or counselling or group therapy or group intervention or trauma informed) OR AB (intervention or treatment or therapy or peer support or support group or trial or psychotherapy or group support or talking therapy or counselling or group therapy or group intervention or trauma informed) OR (MH "Psychotherapy") OR (MH "Self-Help Groups") OR (MH "Peer Group")
S5	TX (stress or anxiety or depression or burnout or worry* or "parent* satisfaction*" or wellbeing or mental health)
S6	TI ((Anxiety or depression or depressive or “obsessive compulsive disorder” or “OCD” or phobia or phobic or mood disorder or anxiety disorder or panic disorder or agoraphobia or internalising problem* or internalising problem* or internalizing problem* or internalizing disorder*)) OR AB ((Anxiety or depression or

	depressive or “obsessive compulsive disorder” or “OCD” or phobia or phobic or mood disorder or anxiety disorder or panic disorder or agoraphobia or internalising problem* or internalising problem* or internalizing problem* or internalizing disorder*) OR (MH "Depressive Disorder") OR (MH "Depressive Disorder, Major") OR (MH "Depressive Disorder, Treatment-Resistant") OR (MH "Dysthymic Disorder") OR (MM "Anxiety Disorders+"))
S6	S1 AND S2 AND S3 AND S4 AND S5 NOT S6

Database: PsycINFO

**Search**	**Term**
S1	TI ((parent or parents or parental or mother or father or care*giver or guardian* or carer* or paternal or maternal)) OR AB ((parent or parents or parental or mother or ather or care*giver or guardian* or carer* or paternal or maternal)) OR MM ("Parents+")
S2	TI ((children or adolescent* or adolescence or youth* or child or teenager* or pediatric* or paediatric* or kid* or teen* or young person or young people or boy* or girl* or juvenile*)) OR AB ((children or adolescent* or adolescence or youth* or child or teenager* or pediatric* or paediatric* or kid* or teen* or young person or young people or boy* or girl* or juvenile*)) OR MH ("Child+") OR MM ("Adolescent")
S3	TI ((attention deficit disorder* or “attention deficit hyperactive disorder* “or “ADHD”) OR AB (attention deficit disorder* or “attention deficit hyperactive disorder* “or “ADHD”)OR MH (“Attention Deficit Disorder with Hyperactivity”) OR (TI ((Eating disorder* or eating problem*)) OR AB ((Eating disorder* or eating problem*) OR MH(“Feeding and Eating Disorders+”) OR (TI ((Emerging personality disorder* or emerging personality problem*)) OR AB ((Emerging personality disorder* or emerging personality problem*)) OR MH (“Personality Disorder+”)) OR (TI ((Externalising disorder* or externalising problem* or externalizing disorder* or externalizing problem*) OR AB (Externalising disorder* or externalising problem* or externalizing disorder* or externalizing problem*) OR TX (“Externalising disorder”)) OR (TI ((Oppositional defiant disorder* or oppositional defiant problem*)) OR AB ((Oppositional defiant disorder* or oppositional defiant problem*) OR (MH“Attention Deficit and Disruptive Behavior Disorders+”)) OR (TI ((Psychos* or psychotic disorder* or psychotic problem*)) OR AB ((Psychos* or psychotic disorder* or psychotic problem*)) OR MH (“Psychotic Disorders+)) OR (TI ((Anxiety or depression or depressive or “obsessive compulsive disorder” or “OCD” or phobia or phobic or mood disorder or anxiety disorder or panic disorder or agoraphobia or internalising problem* or internalising problem* or internalizing problem* or internalizing disorder*)) OR AB ((Anxiety or depression or depressive or “obsessive compulsive disorder” or “OCD” or phobia or phobic or mood disorder or anxiety disorder or panic disorder or agoraphobia or internalising problem* or internalising problem* or internalizing problem* or internalizing disorder*) OR (MH "Depressive Disorder") OR (MH "Depressive Disorder, Major") OR (MH "Depressive Disorder, Treatment-Resistant") OR (MH "Dysthymic Disorder") OR (MM "Anxiety Disorders+")
S4	TI (intervention or treatment or therapy or peer support or support group or trial or psychotherapy or group support or talking therapy or counselling or group therapy or group intervention or trauma informed) OR AB (intervention or treatment or therapy or peer support or support group or trial or psychotherapy or group support or talking therapy or counselling or group therapy or group intervention or trauma informed) OR (MH "Psychotherapy") OR (MH "Self-Help Groups") OR (MH "Peer Group")

S5	TX (stress or anxiety or depression or burnout or worry* or "parent* satisfaction*" or wellbeing or mental health)
S6	TI ((Anxiety or depression or depressive or “obsessive compulsive disorder” or “OCD” or phobia or phobic or mood disorder or anxiety disorder or panic disorder or agoraphobia or internalising problem* or internalising problem* or internalizing problem* or internalizing disorder*)) OR AB ((Anxiety or depression or depressive or “obsessive compulsive disorder” or “OCD” or phobia or phobic or mood disorder or anxiety disorder or panic disorder or agoraphobia or internalising problem* or internalising problem* or internalizing problem* or internalizing disorder*) OR (MH "Depressive Disorder") OR (MH "Depressive Disorder, Major") OR (MH "Depressive Disorder, Treatment-Resistant") OR (MH "Dysthymic Disorder") OR (MM "Anxiety Disorders+"))
S7	S1 AND S2 AND S3 AND S4 AND S5 NOT S6

For peer review only

Database: CINAHL ULTIMATE

Search	Term
S1	TI ((parent or parents or parental or mother or father or care*giver or guardian* or carer* or paternal or maternal)) OR AB ((parent or parents or parental or mother or ather or care*giver or guardian* or carer* or paternal or maternal)) OR MM ("Parents+")
S2	TI ((children or adolescent* or adolescence or youth* or child or teenager* or pediatric* or paediatric* or kid* or teen* or young person or young people or boy* or girl* or juvenile*)) OR AB ((children or adolescent* or adolescence or youth* or child or teenager* or pediatric* or paediatric* or kid* or teen* or young person or young people or boy* or girl* or juvenile*)) OR MH ("Child+") OR MM ("Adolescent")
S3	TI ((attention deficit disorder* or “attention deficit hyperactive disorder* “or “ADHD”) OR AB (attention deficit disorder* or “attention deficit hyperactive disorder* “or “ADHD”)OR MH (“Attention Deficit Disorder with Hyperactivity”) OR (TI ((Eating disorder* or eating problem*)) OR AB ((Eating disorder* or eating problem*) OR MH(“Feeding and Eating Disorders+”) OR (TI ((Emerging personality disorder* or emerging personality problem*)) OR AB ((Emerging personality disorder* or emerging personality problem*)) OR MH (“Personality Disorder+”)) OR (TI ((Externalising disorder* or externalising problem* or externalizing disorder* or externalizing problem*) OR AB (Externalising disorder* or externalising problem* or externalizing disorder* or externalizing problem*) OR TX (“Externalising disorder”)) OR (TI ((Oppositional defiant disorder* or oppositional defiant problem*)) OR AB ((Oppositional defiant disorder* or oppositional defiant problem*) OR (MH“Attention Deficit and Disruptive Behavior Disorders+”)) OR (TI ((Psychos* or psychotic disorder* or psychotic problem*)) OR AB ((Psychos* or psychotic disorder* or psychotic problem*)) OR MH (“Psychotic Disorders+)) OR (TI ((Anxiety or depression or depressive or “obsessive compulsive disorder” or “OCD” or phobia or phobic or mood disorder or anxiety disorder or panic disorder or agoraphobia or internalising problem* or internalising problem* or internalizing problem* or internalizing disorder*)) OR AB ((Anxiety or depression or depressive or “obsessive compulsive disorder” or “OCD” or phobia or phobic or mood disorder or anxiety disorder or panic disorder or agoraphobia or internalising problem* or internalising problem* or internalizing problem* or internalizing disorder*) OR (MH "Depressive Disorder") OR (MH "Depressive Disorder, Major") OR (MH "Depressive Disorder, Treatment-Resistant") OR (MH "Dysthymic Disorder") OR (MM "Anxiety Disorders+")
S4	TI (intervention or treatment or therapy or peer support or support group or trial or psychotherapy or group support or talking therapy or counselling or group therapy

	or group intervention or trauma informed) OR AB (intervention or treatment or therapy or peer support or support group or trial or psychotherapy or group support or talking therapy or counselling or group therapy or group intervention or trauma informed) OR (MH "Psychotherapy") OR (MH "Self-Help Groups") OR (MH "Peer Group")
S5	TX (stress or anxiety or depression or burnout or worry* or "parent* satisfaction*" or wellbeing or mental health)
S6	TI ((Anxiety or depression or depressive or "obsessive compulsive disorder" or "OCD" or phobia or phobic or mood disorder or anxiety disorder or panic disorder or agoraphobia or internalising problem* or internalising problem* or internalizing problem* or internalizing disorder*)) OR AB ((Anxiety or depression or depressive or "obsessive compulsive disorder" or "OCD" or phobia or phobic or mood disorder or anxiety disorder or panic disorder or agoraphobia or internalising problem* or internalising problem* or internalizing problem* or internalizing disorder*) OR (MH "Depressive Disorder") OR (MH "Depressive Disorder, Major") OR (MH "Depressive Disorder, Treatment-Resistant") OR (MH "Dysthymic Disorder") OR (MM "Anxiety Disorders+"))
S6	S1 AND S2 AND S3 AND S4 AND S5 NOT S6

Database: AMED

Search	Term
S1	TI ((parent or parents or parental or mother or father or care*giver or guardian* or carer* or paternal or maternal)) OR AB ((parent or parents or parental or mother or ather or care*giver or guardian* or carer* or paternal or maternal)) OR MM ("Parents+")
S2	TI ((children or adolescent* or adolescence or youth* or child or teenager* or pediatric* or paediatric* or kid* or teen* or young person or young people or boy* or girl* or juvenile*)) OR AB ((children or adolescent* or adolescence or youth* or child or teenager* or pediatric* or paediatric* or kid* or teen* or young person or young people or boy* or girl* or juvenile*)) OR MH ("Child+") OR MM ("Adolescent")
S3	TI ((attention deficit disorder* or "attention deficit hyperactive disorder* "or "ADHD") OR AB (attention deficit disorder* or "attention deficit hyperactive disorder* "or "ADHD")OR MH ("Attention Deficit Disorder with Hyperactivity") OR (TI ((Eating disorder* or eating problem*)) OR AB ((Eating disorder* or eating problem*) OR MH("Feeding and Eating Disorders+") OR (TI ((Emerging personality disorder* or emerging personality problem*)) OR AB ((Emerging personality disorder* or emerging personality problem*)) OR MH ("Personality Disorder+")) OR (TI ((Externalising disorder* or externalising problem* or externalizing disorder* or externalizing problem*) OR AB (Externalising disorder* or externalising problem* or externalizing disorder* or externalizing problem*) OR TX ("Externalising disorder")) OR (TI ((Oppositional defiant disorder* or oppositional defiant problem*)) OR AB ((Oppositional defiant disorder* or oppositional defiant problem*) OR (MH"Attention Deficit and Disruptive Behavior Disorders+")) OR (TI ((Psychos* or psychotic disorder* or psychotic problem*)) OR AB ((Psychos* or psychotic disorder* or psychotic problem*)) OR MH ("Psychotic Disorders+)) OR (TI ((Anxiety or depression or depressive or "obsessive compulsive disorder" or "OCD" or phobia or phobic or mood disorder or anxiety disorder or panic disorder or agoraphobia or internalising problem* or internalising problem* or internalizing problem* or internalizing disorder*)) OR AB ((Anxiety or depression or depressive or "obsessive compulsive disorder" or "OCD" or phobia or phobic or mood disorder or anxiety disorder or panic disorder or agoraphobia or internalising problem* or internalising problem* or internalizing

	problem* or internalizing disorder*) OR (MH "Depressive Disorder") OR (MH "Depressive Disorder, Major") OR (MH "Depressive Disorder, Treatment-Resistant") OR (MH "Dysthymic Disorder") OR (MM "Anxiety Disorders+")
S4	TI (intervention or treatment or therapy or peer support or support group or trial or psychotherapy or group support or talking therapy or counselling or group therapy or group intervention or trauma informed) OR AB (intervention or treatment or therapy or peer support or support group or trial or psychotherapy or group support or talking therapy or counselling or group therapy or group intervention or trauma informed) OR (MH "Psychotherapy") OR (MH "Self-Help Groups") OR (MH "Peer Group")
S5	TX (stress or anxiety or depression or burnout or worry* or "parent* satisfaction*" or wellbeing or mental health)
S6	TI ((Anxiety or depression or depressive or "obsessive compulsive disorder" or "OCD" or phobia or phobic or mood disorder or anxiety disorder or panic disorder or agoraphobia or internalising problem* or internalising problem* or internalizing problem* or internalizing disorder*)) OR AB ((Anxiety or depression or depressive or "obsessive compulsive disorder" or "OCD" or phobia or phobic or mood disorder or anxiety disorder or panic disorder or agoraphobia or internalising problem* or internalising problem* or internalizing problem* or internalizing disorder*) OR (MH "Depressive Disorder") OR (MH "Depressive Disorder, Major") OR (MH "Depressive Disorder, Treatment-Resistant") OR (MH "Dysthymic Disorder") OR (MM "Anxiety Disorders+"))
S7	S1 AND S2 AND S3 AND S4 AND S5 NOT S6

Database: The Cochrane Library (including the Cochrane Database of Systematic Reviews, the Cochrane Central Register of Controlled Trials (CENTRAL), the Database of Abstracts of Reviews of Effects, the Health Technology Assessment Database, and the NHS Economic Evaluation Database)

**Search**	**Term**
S1	TI ((parent or parents or parental or mother or father or care*giver or guardian* or carer* or paternal or maternal)) OR AB ((parent or parents or parental or mother or ather or care*giver or guardian* or carer* or paternal or maternal)) OR MM ("Parents+")
S2	TI ((children or adolescent* or adolescence or youth* or child or teenager* or pediatric* or paediatric* or kid* or teen* or young person or young people or boy* or girl* or juvenile*)) OR AB ((children or adolescent* or adolescence or youth* or child or teenager* or pediatric* or paediatric* or kid* or teen* or young person or young people or boy* or girl* or juvenile*)) OR MH ("Child+") OR MM ("Adolescent"))
S3	attention deficit disorder* or attention deficit hyperactive disorder* or ADHD or Eating disorder* or eating problem* or Emerging personality disorder or emerging personality problem* or Externalising disorder* or externalising problem* or externalizing disorder* or externalizing problem* or Oppositional defiant disorder* or oppositional defiant problem* or Psychos* or psychotic disorder* or psychotic problem* or Anxiety or depression or depressive or obsessive compulsive disorder or OCD or phobia or phobic or mood disorder or anxiety disorder or panic disorder or agoraphobia or internalising problem* or internalising problem* or internalizing problem* or internalizing disorder*
S4	TI (intervention or treatment or therapy or peer support or support group or trial or psychotherapy or group support or talking therapy or counselling or group therapy or group intervention or trauma informed) OR AB (intervention or treatment or

For peer review only

	therapy or peer support or support group or trial or psychotherapy or group support or talking therapy or counselling or group therapy or group intervention or trauma informed) OR (MH "Psychotherapy") OR (MH "Self-Help Groups") OR (MH "Peer Group")
S5	TX (stress or anxiety or depression or burnout or worry* or "parent* satisfaction*" or wellbeing or mental health)
S6	anxiety OR depression OR depressive OR 'obsessive compulsive disorder' OR 'ocd' OR phobia OR phobic OR 'mood disorder' OR 'anxiety disorder':ab,ti OR 'panic disorder' OR agoraphobia OR 'internalising problem*' OR 'internalising disorder*' OR 'internalizing problem*' OR 'internalizing disorder*':ab,ti
S7	S1 AND S2 AND S3 AND S4 AND S5 NOT S6

Database: Web of Science Core Collection

Search	Terms
1	(TI=(parent OR parents OR parental OR mother OR father OR care*giver OR guardian* OR carer* OR paternal OR maternal)) OR (AB=(parent OR parents OR parental OR mother OR father OR care*giver OR guardian* OR carer* OR paternal OR maternal))
2	(TI=(children OR adolescent* OR adolescence OR youth* OR child OR teenager* OR pediatric* OR paediatric* OR kid* OR teen* OR 'young person' OR 'young people' OR boy* OR girl* OR juvenile*)) OR (AB=(children OR adolescent* OR adolescence OR youth* OR child OR teenager* OR pediatric* OR paediatric* OR kid* OR teen* OR 'young person' OR 'young people' OR boy* OR girl* OR juvenile*))
3	(TI=('attention deficit disorder*' OR 'attention deficit hyperactive disorder*' OR adhd OR 'attention deficit disorder with hyperactivity' OR 'eating disorder*' OR 'eating problem*' OR 'feeding and eating disorders' OR 'emerging personality disorder*' OR 'emerging personality problem*' OR 'personality disorder' OR 'externalising disorder*' OR 'externalising problem*' OR 'externalizing disorder*' OR 'externalizing problem*' OR 'oppositional defiant disorder*' OR 'oppositional defiant problem*' OR 'attention deficit and disruptive behavior disorders' OR psychos* OR 'psychotic disorder*' OR 'psychotic problem*' OR anxiety OR depression OR depressive OR 'obsessive compulsive disorder' OR 'ocd' OR phobia OR phobic OR 'mood disorder' OR 'anxiety disorder' OR 'panic disorder' OR agoraphobia OR 'internalising problem*' OR 'internalising disorder*' OR 'internalizing problem*' OR 'internalizing disorder*)) OR (AB=('attention deficit disorder*' OR 'attention deficit hyperactive disorder*' OR adhd OR 'attention deficit disorder with hyperactivity' OR 'eating disorder*' OR 'eating problem*' OR 'feeding and eating disorders' OR 'emerging personality disorder*' OR 'emerging personality problem*' OR 'personality disorder' OR 'externalising disorder*' OR 'externalising problem*' OR 'externalizing disorder*' OR 'externalizing problem*' OR 'oppositional defiant disorder*' OR 'oppositional defiant problem*' OR 'attention deficit and disruptive behavior disorders' OR psychos* OR 'psychotic disorder*' OR 'psychotic problem*' OR anxiety OR depression OR depressive OR 'obsessive compulsive disorder' OR 'ocd' OR phobia OR phobic OR 'mood disorder' OR 'anxiety disorder' OR 'panic disorder' OR agoraphobia OR 'internalising problem*' OR 'internalising disorder*' OR 'internalizing problem*' OR 'internalizing disorder*))
4	(TI=(intervention OR treatment OR therapy OR 'peer support' OR 'support group' OR trial OR psychotherapy OR 'group support' OR 'talking therapy' OR counselling OR 'group therapy' OR 'group intervention' OR 'trauma informed' OR 'self help group' OR 'peer group')) OR (AB=(intervention OR treatment OR therapy OR 'peer support' OR 'support group' OR trial OR psychotherapy OR 'group support' OR 'talking therapy' OR

	counselling OR 'group therapy' OR 'group intervention' OR 'trauma informed' OR 'self help group' OR 'peer group'))
5	(TI=(stress OR anxiety OR depression OR burnout OR worry* OR 'parent* satisfaction*' OR wellbeing OR 'mental health')) OR (AB=(stress OR anxiety OR depression OR burnout OR worry* OR 'parent* satisfaction*' OR wellbeing OR 'mental health'))
6	#5 AND #4 AND #3 AND #2 AND #1
7	(TI=(anxiety OR depression OR depressive OR 'obsessive compulsive disorder' OR 'ocd' OR phobia OR phobic OR 'mood disorder' OR 'anxiety disorder' OR 'panic disorder' OR agoraphobia OR 'internalising problem*' OR 'internalising disorder*' OR 'internalizing problem*' OR 'internalizing disorder*)) OR (AB=(anxiety OR depression OR depressive OR 'obsessive compulsive disorder' OR 'ocd' OR phobia OR phobic OR 'mood disorder' OR 'anxiety disorder' OR 'panic disorder' OR agoraphobia OR 'internalising problem*' OR 'internalising disorder*' OR 'internalizing problem*' OR 'internalizing disorder*))
8	#6 NOT #7

Database: EMBASE

Search	Terms
1	'parent'/exp OR parent OR 'parents'/exp OR parents OR parental OR 'mother'/exp OR mother OR 'father'/exp OR father OR care*giver OR guardian* OR carer* OR paternal OR 'maternal'/exp OR maternal:ti,ab
2	children OR adolescent* OR adolescence OR youth* OR child OR teenager* OR pediatric* OR paediatric* OR kid* OR teen* OR 'young person' OR 'young people' OR boy* OR girl* OR juvenile*:ti,ab
3	'attention deficit disorder*' OR 'attention deficit hyperactive disorder*' OR adhd OR 'attention deficit disorder with hyperactivity' OR 'eating disorder*' OR 'eating problem*' OR 'feeding and eating disorders' OR 'emerging personality disorder*' OR 'emerging personality problem*' OR 'personality disorder' OR 'externalising disorder*' OR 'externalising problem*' OR 'externalizing disorder*' OR 'externalizing problem*' OR 'oppositional defiant disorder*' OR 'oppositional defiant problem*' OR 'attention deficit and disruptive behavior disorders' OR psychos* OR 'psychotic disorder*' OR 'psychotic problem*' OR anxiety OR depression OR depressive OR 'obsessive compulsive disorder' OR 'ocd' OR phobia OR phobic OR 'mood disorder' OR 'anxiety disorder' OR 'panic disorder' OR agoraphobia OR 'internalising problem*' OR 'internalising disorder*' OR 'internalizing problem*' OR 'internalizing disorder*':ti,ab
4	intervention OR treatment OR therapy OR 'peer support' OR 'support group' OR trial OR psychotherapy OR 'group support' OR 'talking therapy' OR counselling OR 'group therapy' OR 'group intervention' OR 'trauma informed' OR 'self help group' OR 'peer group':ti,ab
5	stress OR anxiety OR depression OR burnout OR worry* OR 'parent* satisfaction*' OR wellbeing OR 'mental health':ti,ab
6	#1 AND #2 AND #3 AND #4 AND #5
7	anxiety OR depression OR depressive OR 'obsessive compulsive disorder' OR 'ocd' OR phobia OR phobic OR 'mood disorder' OR 'anxiety disorder':ab,ti OR 'panic disorder' OR agoraphobia OR 'internalising problem*' OR 'internalising disorder*' OR 'internalizing problem*' OR 'internalizing disorder*':ab,ti
8	#6 NOT #7

1

PRISMA-P 2015 Checklist

This checklist has been adapted for use with systematic review protocol submissions to BioMed Central journals from Table 3 in Moher D et al: Preferred reporting items for systematic review and meta-analysis protocols (PRISMA-P) 2015 statement. *Systematic Reviews* 2015 **4**:1

An Editorial from the Editors-in-Chief of *Systematic Reviews* details why this checklist was adapted - **Moher D, Stewart L & Shekelle P:** Implementing PRISMA-P: recommendations for prospective authors. *Systematic Reviews* 2016 **5**:15

Section/topic	#	Checklist item	Information reported		Line number(s)
			Yes	No	
**ADMINISTRATIVE INFORMATION**					
**Title**					
Identification	1a	Identify the report as a protocol of a systematic review	✓		1
Update	1b	If the protocol is for an update of a previous systematic review, identify as such		✓	Not applicable
**Registration**	2	If registered, provide the name of the registry (e.g., PROSPERO) and registration number in the Abstract	✓		41-42
**Authors**					
Contact	3a	Provide name, institutional affiliation, and e-mail address of all protocol authors; provide physical mailing address of corresponding author	✓		4-17
Contributions	3b	Describe contributions of protocol authors and identify the guarantor of the review	✓		292-299
**Amendments**	4	If the protocol represents an amendment of a previously completed or published protocol, identify as such and list changes; otherwise, state plan for documenting important protocol amendments		✓	Not applicable
**Support**					
Sources	5a	Indicate sources of financial or other support for the review	✓		301-303
Sponsor	5b	Provide name for the review funder and/or sponsor	✓		301-303
Role of sponsor/funder	5c	Describe roles of funder(s), sponsor(s), and/or institution(s), if any, in developing the protocol		✓	Not applicable
**INTRODUCTION**					
**Rationale**	6	Describe the rationale for the review in the context of what is already known	✓		68-101
**Objectives**	7	Provide an explicit statement of the question(s) the review will address with reference to participants, interventions, comparators, and outcomes (PICO)	✓		98-101
**METHODS**					

For peer review only

Section/topic	#	Checklist item	Information reported		Line number(s)
			Yes	No	
**Eligibility criteria**	8	Specify the study characteristics (e.g., PICO, study design, setting, time frame) and report characteristics (e.g., years considered, language, publication status) to be used as criteria for eligibility for the review	✓		108-154
**Information sources**	9	Describe all intended information sources (e.g., electronic databases, contact with study authors, trial registers, or other grey literature sources) with planned dates of coverage	✓		156-188
**Search strategy**	10	Present draft of search strategy to be used for at least one electronic database, including planned limits, such that it could be repeated	✓		Supplementary file 1 and 2
STUDY RECORDS					
Data management	11a	Describe the mechanism(s) that will be used to manage records and data throughout the review	✓		203
Selection process	11b	State the process that will be used for selecting studies (e.g., two independent reviewers) through each phase of the review (i.e., screening, eligibility, and inclusion in meta-analysis)	✓		203-208
Data collection process	11c	Describe planned method of extracting data from reports (e.g., piloting forms, done independently, in duplicate), any processes for obtaining and confirming data from investigators	✓		209-238
**Data items**	12	List and define all variables for which data will be sought (e.g., PICO items, funding sources), any pre-planned data assumptions and simplifications	✓		108-154
**Outcomes and prioritization**	13	List and define all outcomes for which data will be sought, including prioritization of main and additional outcomes, with rationale	✓		140-146
**Risk of bias in individual studies**	14	Describe anticipated methods for assessing risk of bias of individual studies, including whether this will be done at the outcome or study level, or both; state how this information will be used in data synthesis	✓		209-238
DATA					
**Synthesis**	15a	Describe criteria under which study data will be quantitatively synthesized	✓		239-276
	15b	If data are appropriate for quantitative synthesis, describe planned summary measures, methods of handling data, and methods of combining data from studies, including any planned exploration of consistency (e.g., I^2, Kendall's tau)	✓		239-276
	15c	Describe any proposed additional analyses (e.g., sensitivity or subgroup analyses, meta-regression)	✓		239-276
	15d	If quantitative synthesis is not appropriate, describe the type of summary planned	✓		239-276
**Meta-bias(es)**	16	Specify any planned assessment of meta-bias(es) (e.g., publication bias across studies, selective reporting within studies)	✓		254-256
**Confidence in cumulative evidence**	17	Describe how the strength of the body of evidence will be assessed (e.g., GRADE)	✓		209-238

BioMed Central
The Open Access Publisher

For peer review only - http://bmjopen.bmj.com/site/about/guidelines.xhtml

BMJ Open

A systematic review protocol of interventions to support parents and carers of young people with mental health difficulties

Journal:	*BMJ Open*
Manuscript ID	bmjopen-2023-073940.R1
Article Type:	Protocol
Date Submitted by the Author:	17-May-2023
Complete List of Authors:	Martin, Faith; Cardiff University<br>Dahmash, Dania; Coventry University<br>Tsang, Anthony; King's College London<br>Glover, Sarah; Parental Minds Community Interest Company<br>Duncan, Charlie; British Association for Counselling and Psychotherapy<br>Halligan, Sarah; University of Bath, Department of Psychology
**Primary Subject Heading**:	Mental health
Secondary Subject Heading:	Mental health, Paediatrics
Keywords:	MENTAL HEALTH, Systematic Review, PUBLIC HEALTH, Adolescent

SCHOLARONE™
Manuscripts

A systematic review protocol of interventions to support parents and carers of young people with mental health difficulties

AUTHORS

Dr Faith Martin. School of Psychology, Cardiff University, 70 Park Pl, Cardiff CF10 3AS. Email martinf8@cardiff.ac.uk

Dr Dania Dahmash. Coventry University, Centre for Intelligent Healthcare, Coventry University, Richard Crossman Building, Jordan Well, Coventry CV1 5RW, UK. Email: ad9819@coventry.ac.uk

Anthony Tsang. King's Technology Evaluation Centre, London Institute for Healthcare Engineering, King's College London. 1 Lambeth Palace Rd, South Bank, London, SE1 7EU, UK. Email: anthony.t@live.co.uk.

Mrs Sarah Glover. Parental Minds Community Interest Company, UK. Email: parentplus@parentalminds.org.uk

Charlie Duncan. Research Department, BACP House, 15 St John's Business Park, Lutterworth, LE17 4HB Email charlie.duncan@bacp.co.uk

Professor Sarah Halligan. University of Bath, Department of Psychology, University of Bath, Claverton Down Campus, Bath BA2 7AY, UK. Email: slh54@bath.ac.uk

CORRESPONDING AUTHOR:

Dr Dania Dahmash. Coventry University, Centre for Intelligent Healthcare, Coventry University, Richard Crossman Building, Jordan Well, Coventry CV1 5RW, UK. Email ad9818@coventry.ac.uk

For peer review only

Abstract

Introduction: Globally 8-14% of children and young people (CYP) have a diagnosable mental health condition, many of whom receive no formal interventions. Parents/carers of CYP experience stress and distress owing to the mental health difficulties encountered by their CYP due to the lack of resources and support. Currently, little is known about 1) the content of interventions developed to support parents/carers nor 2) how effective interventions are at improving parents'/carers' wellbeing. The planned review aims to address these two gaps.

Method and analysis: A systematic review will be conducted to identify any study that describes an intervention aiming at least in part to support parents/carers with the impact of CYP (5-18 years) mental health difficulties, and to review any randomised-controlled trials (RCTs) of these interventions. The following databases will be searched: MEDLINE; PsycINFO; CINAHL; AMED; EMBASE; Web of Science Core Collection and Cochrane Library CENTRAL, without and limitations applied. Analysis of the content of interventions will be structured using the Template for Intervention Description and Replication checklist as a framework. The effect of any RCTs on parents'/carers' outcomes (including wellbeing, satisfaction with parenting, mental health) will be extracted and assessed using the Cochrane Risk-of-Bias Tool. Data will be synthesised narratively, with meta-analysis of RCT results, if appropriate.

Ethical consideration and dissemination: The protocol has been registered on PROSPERO under the registration number: CRD42022344453 and approved by Coventry University Ethical Committee (reference number: P139611). Results will be shared in academic publications and in accessible formats using social media and public webinars.

Keywords:

For peer review only

Children; young people; mental health; parent; carers; interventions; parental needs; systematic review

Strengths and limitations of this study

1- Narrative analysis will be used to analysed the intervention components which is a major strength, providing insight into the details of what the interventions reviewed cover.

2- Primary screening of the articles, data extraction and quality assessment will be performed independently by two persons with extensive experience in systematic review methodology, to minimise the probability of personal biases.

3- The review follows a rigorous method and the results of the review will be reported in accordance with Preferred Reporting Items for Systematic Review and Meta-Analysis reporting guidelines.

4- This systematic review protocol reduces the possibility of duplication due to the transparency of the methods and processes that will be used; in addition, it reduces possible biases and allows for peer review.

5- In this study, databases in languages other than English (French, German, Chinese, etc) will not be searched or included. This limitation may cause language bias.

For peer review only

INTRODUCTION

Mental health conditions amongst children and young people (CYP) are increasingly common: globally around 8% of 5-9 year olds and 14% of 10-18 years old have a diagnosable mental health condition (1). In the UK, approximately one in six school-aged children have a mental health problem (2). Demand is outstripping service provision, with lengthy waiting times and only around a quarter (27%) of CYP who need mental health support in the UK are receiving it (3). This may place greater pressure on families to manage the difficulties on their own. Therefore, parents and carers (hereafter "parents") are often experiencing stress and distress in relation to their CYP's mental health difficulties, including depression, managing stigma, anxiety, and work and financial pressures (4-6).

Policy recommendations include seeing parents as partners in their CYP's care (7), however little attention is paid to the parent`s own needs. The World Health Organization's (WHO) report describes the importance of supporting parents` mental health, however, focuses on parents with pre-existing mental health conditions and new parents (1). There is a reference to the needs of parents of CYP with developmental delays or difficulties, highlighting that they are likely to experience high levels of distress. Whilst clearly vital, there is a need to also support parents with the understandable distress of having a CYP with a mental health difficulty.

Interventions have been developed that include parents to address CYP mental health. Parent training, family therapy, and parent-co-delivered/led cognitive behavioural therapy all, to some extent, involve the parent (8-10). However, the focus is frequently not on the parents' wellbeing, nor on addressing the impact of CYP mental health difficulties on the parent. The content of interventions (or elements of interventions) that specifically aim to support parents has not yet been identified or summarised. This is important, as identification of the content

For peer review only

can support the evaluation of effectiveness, for example, interventions may be focusing on just one element of parental distress by providing information, and thus not necessarily achieving good outcomes. The content of the interventions can be compared to the needs that have been identified for parents/carers(11).

Given the lack of policy guidelines and existing evidence synthesis, this systematic review protocol outlines the process for identifying literature which addresses the following research questions:

1- What is the content of interventions or intervention components that have been designed to address parents' wellbeing in the context of CYP mental health difficulties

2- How effective are these interventions in terms of improving parents' wellbeing outcomes, family wellbeing and mental health service utilisation?

METHOD AND ANALYSIS

The study has been registered in the International Prospective Register of Systematic Reviews (PROSPERO: CRD42022344453). This protocol follows the Preferred Reporting Items for Systematic Review and Meta-Analyses for Protocols (PRISMA-P) 2015 guidelines (12). The completed systematic review will adhere to the reporting guidelines of PRISMA-2020 (13).

Study selection criteria

Participants

The participants to be included in this review will be parents of CYP (aged 5-18) who have a diagnosis of a mental health condition. This age range reflects the upper limit of most CYP-specific mental health services and covers CYP before they have gone to university, therefore are more likely to still be at home with parents, with the lower limit relating to the youngest

For peer review only

age of onset for most conditions (14). To be included, the majority (>50%) of the CYP must be within that age range. The CYP must have at least one diagnosed mental health condition from depression, anxiety disorders, psychoses, oppositional defiant and other externalising disorders, labels of emerging personality disorders, eating disorders, and attention deficit (hyperactive) disorders. "Parents" covers all adults (aged 18 years and over) in a formal parenting role, including biological parents, step-parents, other relatives assuming a parenting role, non-biological and adoptive parents, foster carers, and other adults in legal guardian roles. The review does not cover parents/carers of CYP with special educational needs, including autism spectrum, as there are existing reviews and services and needs are different (15). Where the focus is on CYP with mental health difficulties and within that includes less than 50% CYP with developmental/special educational needs, studies would be included. Study samples can be drawn from any setting, for example, supporting parents of CYP on waiting lists, or receiving care through voluntary or statutory services, providing that the CYP has a diagnosed condition.

Intervention

The intervention must include a component specifically designed to meet the parents'/carers' needs – be that information, emotional or social support. Interventions that train parents/carers to provide therapy or behavioural management techniques, where the focus of the intervention is solely on changing CYP outcomes, will not be included. Interventions that include therapy for the child but also support for parents/carers will be included. However, for research question two, the intervention need not be described in full in the study however must provide sufficient evidence (e.g. brief description, reference to a study that does describe the intervention) of relevance to the review.

Comparison

For peer review only

This only applies to research question two. The comparison can be with any control group, including treatment as usual or active controls.

Outcomes

For research question two, outcomes must be measured using validated tools only. Relevant outcomes are parental mental health (e.g., depression, anxiety, stress); parent-CYP relationships; satisfaction with parenting; parenting self-efficacy; family wellbeing; or parent/carer health service utilisation. Search terms relating to the outcomes are used to identify studies for research question one and also, to identify interventions that address our outcomes of interest.

Study design

For research question one, relating to the content of interventions, studies must describe an intervention with at least one component to support parents'/carers' needs or wellbeing. Studies can be of any design and do not need to report any intervention outcomes. For research question two, randomised controlled trials (RCTs) will be included and assessed following Cochrane Reviewing guidelines (16). We will also include a narrative synthesis of studies that describe an intervention and its early-stage testing (e.g. feasibility trials, case series).

Search strategy

The following bibliographic databases were searched: MEDLINE(EBSCO); PsycINFO (Ovid); CINAHL Ultimate (EBSCO); AMED(EBSCO); EMBASE(Ovid); Web of Science Core Collection; Cochrane Library databases including: Cochrane Database of Systematic Reviews, Cochrane Central Register of Controlled Trials, Database of Abstracts of Reviews of

For peer review only

Effects, Health Technology Assessment Database, and NHS Economic Evaluation Database. No filters were applied to the search.

The reference lists of any identified systematic reviews will be screened for additional studies not captured by the database search. Supplementary searches will be performed in the form of forward and backward citations using Citationchaser to potentially identify additional studies(17).

The search terms will include five blocks of terms, all combined with "AND". These terms cover:

1. Parents or carers
2. Children and young people
3. Mental health problems
4. Intervention or treatment or therapy or peer support psychotherapy or counselling or group therapy.
5. Outcomes of interest including stress, anxiety, depression, burnout, or parenting satisfaction

A scoping search was conducted using the above listed-terms, in which (3,827,850) records were identified. This scoping search was used to refine search terms, particularly the use of subject headings. To manage the downloading of records and the workload thereafter, two complementary search strategies will be used (supplementary files 1 and 2). The first search strategy will focus on studies relating to CYP anxiety and/or depression. This is owing to these conditions being very common and representing a large portion of the overall records from the scoping search. The second search strategy then will identify studies linked to all other included CYP mental health difficulties. All criteria for inclusion/exclusion and data extraction will be conducted in the same way – this split is merely to manage the search results rather than conduct separate reviews. (If search 1 identifies studies that extend beyond anxiety and or depression to other included conditions, they will be included during screening).

For peer review only

The planned analysis includes considering results in relation to CYP difficulty, as well as examining similarities/differences in intervention content and efficacy across CYP difficulties.

Patient and Public involvement

The scope of the review has been defined through discussion with parents of CYP with mental health difficulties (including with co-author SG's group "Parental Minds"). This PPI engagement helped to design the study, refine the scope, and specify the protocol, including the exclusion of autistic spectrum disorders and development difficulties from this review.

During the review, we will consult with a group of parents/carers as needed on decisions made, for example, the relevance of interventions to parents' needs and thus inclusion in the review. Initial synthesis and interpretation of findings will be shared with our PPI group for discussion and refinement. PPI involvement, particularly in writing interpretation and recommendations, is planned for the writing of all outputs, the design of non-academic papers, and wider dissemination activities.

DATA COLLECTION AND ANALYSIS

Selection process

Rayyan software will be used to manage records (18, 19). This facilitates blind decisions for inclusion/exclusion. Inclusion/exclusion criteria will be reviewed by stakeholders. A two-step process will be used. First, all titles and abstracts will be screened against the inclusion/exclusion criteria by two reviewers independently. Second, reports will be obtained for short-listed records and inclusion/exclusion will be conducted by two reviewers independently. Any discrepancies at either step will be resolved by a third reviewer if needed.

Data extraction and quality appraisal

For peer review only

A data extraction form will be created and piloted by at least two reviewers. Data will be extracted by two reviewers (D.D and A.T) and all data will be checked by a third reviewer. Data will be extracted into an Excel sheet. Discrepancies will be resolved by discussion with a third reviewer. For both questions extracted data will include, study characteristics (author, study design, setting, aim of the study and sample size) and population characteristics (CYP age, Parents/carers age, ethnicity and relationship to CYP).

For research question one, detailed information about the types of interventions developed will be extracted independently by two reviewers using the Template for Intervention Description and Replication (TIDieR) checklist as the basis to assess the completeness of the descriptions, therefore in relation to this research question, this also covers the quality appraisal(20). This checklist includes: 1) descriptions of needs addressed, 2) aims of the intervention, 3) intervention content, 4) materials used, 5) inclusion criteria, 6) number and duration of sessions, 7) who delivers content and how, 8) where/what modality, 9) personalisation or tailoring of the intervention, 10) underpinning theory, or approach. Information about implementation, including structure and content of training and supervision, and resource requirements will be included. If information is not specifically provided in a study, we will consult protocol and intervention design reports, and contact authors for further information as necessary. Where studies describe interventions for parents/carers and children, data will be extracted relating to the intervention or components for parents/carers. Data will also be extracted relating to any proposed mechanisms of how the intervention may operate.

For research question two, details of the participant characteristics, the intervention itself (covered in research question one), comparison group(s), and details of outcomes (e.g. validated measures of parent/carer mental health (depression, anxiety, stress); parent-CYP relationships; satisfaction with parenting; family wellbeing; and parent/carer health service utilisation) will be extracted. CYP MH service utilisation will also be extracted, if reported in

For peer review only

parent/carer only interventions, to elucidate any impact on this important service outcome. Comparisons between intervention and control follow-up data points will be extracted, with effect sizes, to indicate the impact of the intervention in comparison to control. Quality will be appraised using the Cochrane Risk of Bias Tool(Version 2) (16).

Data synthesis

For research question one, the aim is to describe in detail and categorise approaches to interventions to support parents/carers, mapping these onto parent/carer needs and describing underpinning theoretical approaches. No established a priori framework to categorise these interventions exists. Content analysis will be used to synthesise the data (21-23), within each data field from the TIDieR checklist. For example, codes for the underpinning theories will be created. Two reviewers will independently analyse and code the data, with a discussion of disagreements taken to a third reviewer, parent/carer working group, or wider stakeholder group if required. Content analysis of intervention details will be tabulated, capturing the frequency of inclusion of different intervention content and other intervention details. Where multiple studies describe the same intervention, extracted data will be synthesised into a single entry for that intervention. Findings will also be grouped by CYP diagnosis and compared across different diagnoses.

For research question two, Cochrane systematic review of interventions methods will be followed (16). Mean differences will be used for continuous measures and for outcomes where different measures are used (e.g., anxiety, depression), standardised mean differences. In the absence of significant heterogeneity, and if there are sufficient studies, meta-analysis will be considered using random effects models using RevMan 5(24). This will be conducted where possible in relation to each outcome variable group (e.g. parents' depression, parents' stress). In the presence of significant heterogeneity a narrative synthesis approach will be followed

For peer review only

using Cochrane guidance (25). If there are sufficient studies, stratified analyses will be conducted by CYP diagnoses, intervention type, and active versus inactive comparators. Sensitivity analyses will be performed excluding studies at high risk of bias. Publication bias will be explored using funnel plots if there are sufficient studies. Identified as important in PPI discussions, one reviewer will review the summaries, and comment on the extent to which the specifically identified outcome measures are patient-centred. A vital element of the analysis will be to compare the outcomes of interventions in relation to different CYP mental health difficulties. Interventions that do not select participants based on CYP mental health difficulty will be compared to those for specific problems, as well as comparisons between specific CYP mental health difficulties. If possible, these sub-group analyses will comprise of meta-analyses of studies by CYP mental health difficulty, however, if meta-analyses are not possible (for example, if high levels of heterogeneity as measured by I^2), a narrative synthesis will consider the potential relevance of CYP mental health difficulty type to on reported outcomes, and considering any reported cross-cutting factors such a risk to CYP and duration of CYP mental health difficulty. Sub-group analysis will also be considered to examine differences in relation to whether CYP is receiving or awaiting care; whether care is via statutory or non-statutory services; and by intervention type if there are clear differences in theoretical approaches to the interventions.

ETHICS AND DISSEMINATION

This study was approved by Coventry University ethical committee, reference number: P139611.

Dissemination will use multiple channels such as YouTube and social media, in addition to traditional academic outputs. We will disseminate findings via all our collaborators and

For peer review only

partners in the NHS and charitable sectors to use their networks to share the findings widely. We particularly seek to reach UK policymakers and clinicians using forums such as the Future NHS Collaboration platform. Parents/carers involved in the project will be sent the final outputs via email links/hard copies.

COMPETING INTERESTS

All authors have completed the ICMJE uniform disclosure form. SG is an author and the director of Parental Minds, which provide services to the parent of children with mental health. The authors have no competing interests to declare.

AUTHORS' CONTRIBUTIONS

FM created the idea and led the project, conduced the databases searches and contributing to all stages. FM and DD prepared the manuscript draft. SG, CD, AT and SH contributed to the writing and the reviewing of and the manuscript. Study selection and data collection will be conducted by FM, DD and AT. FM, DD, SG, CD, AT will be involved in the synthesis and interpretation of the data. FM, DD, SG, CD, AT, and SH along with the parent/carer panel will be involved in the final drafting and dissemination of findings. FM, DD, SG, CD, AT, and SH read and approved the final manuscript.

FUNDING

This work was supported by the National Institute of Health Research (NIHR); grant number: NIHR20302.

For peer review only

ACKNOWLEDGEMENTS

We would like to acknowledge and thank the members of the PPI group who have provided invaluable input to date.

LIST OF ABBREVIATIONS

CAMHS – Child and Adolescent Mental Health Service

MH-Mental health

CYP- Child and Young Person

TIDieR- Template for intervention description and replication

For peer review only

REFERENCES

1. WHO. World mental health report: transforming mental health for all. World Health Organization (WHO), accessed January 2023 from https://www.who.int/publications/i/item/9789240049338; 2022.

2. Newlove-Delgado T, Williams T, Robertson K, McManus S, Sadler K, Vizard T, et al. Mental Health of Children and Young People in England 2021-wave 2 follow up to the 2017 survey. 2021.

3. Grimm F, Alcock B, Butler J, Fernandez Crespo R, Davies A, Peytrignet S, et al. Improving children and young people's mental health services. 2022.

4. Shenaar-Golan V, Wald N, Yatzkar U. Parenting a Child with Mental Health Problems: the Role of Self-Compassion. Mindfulness. 2021;12(11):2810-9.

5. Whitlock J, Lloyd-Richardson E, Fisseha F, Bates T. Parental Secondary Stress: The Often Hidden Consequences of Nonsuicidal Self-Injury in Youth. Journal of clinical psychology. 2018;74(1):178-96.

6. Gerkensmeyer JE, Perkins SM, Day J, Austin JK, Scott EL, Wu J. Maternal depressive symptoms when caring for a child with mental health problems. Journal of Child and Family Studies. 2011;20(5):685-95.

7. Care Quality Commission. Review of children and young people's mental health services. Care Quality Commission, London. 2017.

8. Reyno SM, McGrath PJ. Predictors of parent training efficacy for child externalizing behavior problems – a meta-analytic review. Journal of Child Psychology and Psychiatry. 2006;47(1):99-111.

9. Jewell T, Blessitt E, Stewart C, Simic M, Eisler I. Family therapy for child and adolescent eating disorders: a critical review. Family Process. 2016;55(3):577-94.

10. Lawrence P, Parkinson M, Jasper B, Creswell C, Halligan S. Supporting parents of children and young people with anxiety and depressive disorders, an opportunity not to be missed: A scoping review. Lancet Psychiatry. 2020;in press.

For peer review only

11. Armitage S, Parkinson M, Halligan S, Reynolds SJJoC, Studies F. Mothers' experiences of having an adolescent child with depression: An interpretative phenomenological analysis. 2020;29:1617-29.

12. Moher D, Shamseer L, Clarke M, Ghersi D, Liberati A, Petticrew M, et al. Preferred reporting items for systematic review and meta-analysis protocols (PRISMA-P) 2015 statement. 2015;4(1):1-9.

13. Page MJ, McKenzie JE, Bossuyt PM, Boutron I, Hoffmann TC, Mulrow CD, et al. The PRISMA 2020 statement: an updated guideline for reporting systematic reviews. 2021;88:105906.

14. Kessler RC, Berglund P, Demler O, Jin R, Merikangas KR, Walters EE. Lifetime Prevalence and Age-of-Onset Distributions of DSM-IV Disorders in the National Comorbidity Survey Replication. Archives of General Psychiatry. 2005;62(6):593-602.

15. Frantz R, Hansen SG, Machalicek W. Interventions to promote well-being in parents of children with autism: A systematic review. Review Journal of Autism and Developmental Disorders. 2018;5:58-77.

16. Higgins JP, Thomas J, Chandler J, Cumpston M, Li T, Page MJ, et al. Cochrane handbook for systematic reviews of interventions: John Wiley & Sons; 2019.

17. Haddaway N, Grainger M, Gray CJZ, Feb. citationchaser: An R package and Shiny app for forward and backward citations chasing in academic searching. 2021;16.

18. Ouzzani M, Hammady H, Fedorowicz Z, Elmagarmid AJSr. Rayyan—a web and mobile app for systematic reviews. 2016;5:1-10.

19. Covidence. Covidence systematic review software, Veritas Health Innovation.

20. Hoffmann TC, Glasziou PP, Boutron I, Milne R, Perera R, Moher D, et al. Better reporting of interventions: template for intervention description and replication (TIDieR) checklist and guide. 2014;348.

21. Ash T, Agaronov A, Young TL, Aftosmes-Tobio A, Davison KKJIJoBN, Activity P. Family-based childhood obesity prevention interventions: a systematic review and quantitative content analysis. 2017;14(1):1-12.

For peer review only

22. Marston N, Stavnes K, Van Loon LM, Drost LM, Maybery D, Mosek A, et al. A content analysis of Intervention Key Elements and Assessments (IKEA): What's in the black box in the interventions directed to families where a parent has a mental illness? 2016;37(2):112-28.

23. Hsieh H-F, Shannon SEJQhr. Three approaches to qualitative content analysis. 2005;15(9):1277-88.

24. Manager R. Version 5.4, The Cochrane Collaboration. The Nordic Cochrane Centre Copenhagen, Denmark; 2020.

25. Campbell M, McKenzie JE, Sowden A, Katikireddi SV, Brennan SE, Ellis S, et al. Synthesis without meta-analysis (SWiM) in systematic reviews: reporting guideline. 2020;368.

For peer review only

Search terms used for depression and anxiety studies:

Database: Medline

Search	Term
S1 (Parent)	TI ((parent or parents or parental or mother or father or care*giver or guardian* or carer* or paternal or maternal)) OR AB ((parent or parents or parental or mother or ather or care*giver or guardian* or carer* or paternal or maternal)) OR MM ("Parents+")
S2 (children)	TI ((children or adolescent* or adolescence or youth* or child or teenager* or pediatric* or paediatric* or kid* or teen* or young person or young people or boy* or girl* or juvenile*)) OR AB ((children or adolescent* or adolescence or youth* or child or teenager* or pediatric* or paediatric* or kid* or teen* or young person or young people or boy* or girl* or juvenile*)) OR MH ("Child+") OR MM ("Adolescent")
S3 (Mental Health)	TI ((Anxiety or depression or depressive or "obsessive compulsive disorder" or "OCD" or phobia or phobic or mood disorder or anxiety disorder or panic disorder or agoraphobia or internalising problem* or internalising problem* or internalizing problem* or internalizing disorder*)) OR AB ((Anxiety or depression or depressive or "obsessive compulsive disorder" or "OCD" or phobia or phobic or mood disorder or anxiety disorder or panic disorder or agoraphobia or internalising problem* or internalising problem* or internalizing problem* or internalizing disorder*) OR (MH "Depressive Disorder") OR (MH "Depressive Disorder, Major") OR (MH "Depressive Disorder, Treatment-Resistant") OR (MH "Dysthymic Disorder") OR (MM "Anxiety Disorders+"))
S4 (Intervention)	TI (intervention or treatment or therapy or peer support or support group or trial or psychotherapy or group support or talking therapy or counselling or group therapy or group intervention or trauma informed) OR AB (intervention or treatment or therapy or peer support or support group or trial or psychotherapy or group support or talking therapy or counselling or group therapy or group intervention or trauma informed) OR (MH "Psychotherapy") OR (MH "Self-Help Groups") OR (MH "Peer Group")
S5 (outcome)	TX (stress or anxiety or depression or burnout or worry* or "parent* satisfaction*" or wellbeing or mental health)
S6	S1 AND S2 AND S3 AND S4 AND S5

Database: PsycINFO

Search	Term
S1 (Parent)	TI ((parent or parents or parental or mother or father or care*giver or guardian* or carer* or paternal or maternal)) OR AB ((parent or parents or parental or mother or ather or care*giver or guardian* or carer* or paternal or maternal)) OR MM ("Parents+")
S2 (children)	TI ((children or adolescent* or adolescence or youth* or child or teenager* or pediatric* or paediatric* or kid* or teen* or young person or young people or boy* or girl* or juvenile*)) OR AB ((children or adolescent* or adolescence or youth* or child or teenager* or pediatric* or paediatric* or kid* or teen* or young person or young people or boy* or girl* or juvenile*)) OR MH ("Child+") OR MM ("Adolescent")
S3 (Mental Health)	TI ((Anxiety or depression or depressive or "obsessive compulsive disorder" or "OCD" or phobia or phobic or mood disorder or anxiety disorder or panic disorder or agoraphobia or internalising problem* or internalising problem* or internalizing problem* or internalizing disorder*)) OR AB ((Anxiety or depression or depressive or "obsessive compulsive disorder" or "OCD" or phobia or phobic or

For peer review only

	mood disorder or anxiety disorder or panic disorder or agoraphobia or internalising problem* or internalising problem* or internalizing problem* or internalizing disorder*) OR (MH "Depressive Disorder") OR (MH "Depressive Disorder, Major") OR (MH "Depressive Disorder, Treatment-Resistant") OR (MH "Dysthymic Disorder") OR (MM "Anxiety Disorders+"))
S4 (Intervention)	TI (intervention or treatment or therapy or peer support or support group or trial or psychotherapy or group support or talking therapy or counselling or group therapy or group intervention or trauma informed) OR AB (intervention or treatment or therapy or peer support or support group or trial or psychotherapy or group support or talking therapy or counselling or group therapy or group intervention or trauma informed) OR (MH "Psychotherapy") OR (MH "Self-Help Groups") OR (MH "Peer Group")
S5 (outcome)	TX (stress or anxiety or depression or burnout or worry* or "parent* satisfaction*" or wellbeing or mental health)
S6	S1 AND S2 AND S3 AND S4 AND S5

Database: CINAHL ULTIMATE

**Search**	**Term**
S1 (Parent)	TI ((parent or parents or parental or mother or father or care*giver or guardian* or carer* or paternal or maternal)) OR AB ((parent or parents or parental or mother or ather or care*giver or guardian* or carer* or paternal or maternal)) OR MM ("Parents+")
S2 (children)	TI ((children or adolescent* or adolescence or youth* or child or teenager* or pediatric* or paediatric* or kid* or teen* or young person or young people or boy* or girl* or juvenile*)) OR AB ((children or adolescent* or adolescence or youth* or child or teenager* or pediatric* or paediatric* or kid* or teen* or young person or young people or boy* or girl* or juvenile*)) OR MH ("Child+") OR MM ("Adolescent")
S3 (Mental Health)	TI ((Anxiety or depression or depressive or “obsessive compulsive disorder” or “OCD” or phobia or phobic or mood disorder or anxiety disorder or panic disorder or agoraphobia or internalising problem* or internalising problem* or internalizing problem* or internalizing disorder*)) OR AB ((Anxiety or depression or depressive or “obsessive compulsive disorder” or “OCD” or phobia or phobic or mood disorder or anxiety disorder or panic disorder or agoraphobia or internalising problem* or internalising problem* or internalizing problem* or internalizing disorder*) OR (MH "Depressive Disorder") OR (MH "Depressive Disorder, Major") OR (MH "Depressive Disorder, Treatment-Resistant") OR (MH "Dysthymic Disorder") OR (MM "Anxiety Disorders+"))
S4 (Intervention)	TI (intervention or treatment or therapy or peer support or support group or trial or psychotherapy or group support or talking therapy or counselling or group therapy or group intervention or trauma informed) OR AB (intervention or treatment or therapy or peer support or support group or trial or psychotherapy or group support or talking therapy or counselling or group therapy or group intervention or trauma informed) OR (MH "Psychotherapy") OR (MH "Self-Help Groups") OR (MH "Peer Group")
S5 (outcome)	TX (stress or anxiety or depression or burnout or worry* or "parent* satisfaction*" or wellbeing or mental health)
S6	S1 AND S2 AND S3 AND S4 AND S5

Database: AMED

Search	Term
S1 (Parent)	TI ((parent or parents or parental or mother or father or care*giver or guardian* or carer* or paternal or maternal)) OR AB ((parent or parents or parental or mother or ather or care*giver or guardian* or carer* or paternal or maternal)) OR MM ("Parents+")
S2 (children)	TI ((children or adolescent* or adolescence or youth* or child or teenager* or pediatric* or paediatric* or kid* or teen* or young person or young people or boy* or girl* or juvenile*)) OR AB ((children or adolescent* or adolescence or youth* or child or teenager* or pediatric* or paediatric* or kid* or teen* or young person or young people or boy* or girl* or juvenile*)) OR MH ("Child+") OR MM ("Adolescent")
S3 (Mental Health)	TI ((Anxiety or depression or depressive or "obsessive compulsive disorder" or "OCD" or phobia or phobic or mood disorder or anxiety disorder or panic disorder or agoraphobia or internalising problem* or internalising problem* or internalizing problem* or internalizing disorder*)) OR AB ((Anxiety or depression or depressive or "obsessive compulsive disorder" or "OCD" or phobia or phobic or mood disorder or anxiety disorder or panic disorder or agoraphobia or internalising problem* or internalising problem* or internalizing problem* or internalizing disorder*) OR (MH "Depressive Disorder") OR (MH "Depressive Disorder, Major") OR (MH "Depressive Disorder, Treatment-Resistant") OR (MH "Dysthymic Disorder") OR (MM "Anxiety Disorders+"))
S4 (Intervention)	TI (intervention or treatment or therapy or peer support or support group or trial or psychotherapy or group support or talking therapy or counselling or group therapy or group intervention or trauma informed) OR AB (intervention or treatment or therapy or peer support or support group or trial or psychotherapy or group support or talking therapy or counselling or group therapy or group intervention or trauma informed) OR (MH "Psychotherapy") OR (MH "Self-Help Groups") OR (MH "Peer Group")
S5 (outcome)	TX (stress or anxiety or depression or burnout or worry* or "parent* satisfaction*" or wellbeing or mental health)
S7	S1 AND S2 AND S3 AND S4 AND S5

Database: The Cochrane Library (including the Cochrane Database of Systematic Reviews, the Cochrane Central Register of Controlled Trials (CENTRAL), the Database of Abstracts of Reviews of Effects, the Health Technology Assessment Database, and the NHS Economic Evaluation Database)

Search	Term
S1 (Parent)	TI ((parent or parents or parental or mother or father or care*giver or guardian* or carer* or paternal or maternal)) OR AB ((parent or parents or parental or mother or ather or care*giver or guardian* or carer* or paternal or maternal)) OR MM ("Parents+")
S2 (children)	TI ((children or adolescent* or adolescence or youth* or child or teenager* or pediatric* or paediatric* or kid* or teen* or young person or young people or boy* or girl* or juvenile*)) OR AB ((children or adolescent* or adolescence or youth* or child or teenager* or pediatric* or paediatric* or kid* or teen* or young person or young people or boy* or girl* or juvenile*)) OR MH ("Child+") OR MM ("Adolescent"))
S3 (Mental Health)	TI ((Anxiety or depression or depressive or "obsessive compulsive disorder" or "OCD" or phobia or phobic or mood disorder or anxiety disorder or panic disorder or agoraphobia or internalising problem* or internalising problem* or internalizing problem* or internalizing disorder*)) OR AB ((Anxiety or depression or

	depressive or "obsessive compulsive disorder" or "OCD" or phobia or phobic or mood disorder or anxiety disorder or panic disorder or agoraphobia or internalising problem* or internalising problem* or internalizing problem* or internalizing disorder*) OR (MH "Depressive Disorder") OR (MH "Depressive Disorder, Major") OR (MH "Depressive Disorder, Treatment-Resistant") OR (MH "Dysthymic Disorder") OR (MM "Anxiety Disorders+"))
S4 (Intervention)	TI (intervention or treatment or therapy or peer support or support group or trial or psychotherapy or group support or talking therapy or counselling or group therapy or group intervention or trauma informed) OR AB (intervention or treatment or therapy or peer support or support group or trial or psychotherapy or group support or talking therapy or counselling or group therapy or group intervention or trauma informed) OR (MH "Psychotherapy") OR (MH "Self-Help Groups") OR (MH "Peer Group")
S5 (outcome)	TX (stress or anxiety or depression or burnout or worry* or "parent* satisfaction*" or wellbeing or mental health)
S7	S1 AND S2 AND S3 AND S4 AND S5

Database: Web of Science Core Collection

Search	Terms
1	(TI=(parent OR parents OR parental OR mother OR father OR care*giver OR guardian* OR carer* OR paternal OR maternal)) OR (AB=(parent OR parents OR parental OR mother OR father OR care*giver OR guardian* OR carer* OR paternal OR maternal))
2	(TI=(children OR adolescent* OR adolescence OR youth* OR child OR teenager* OR pediatric* OR paediatric* OR kid* OR teen* OR 'young person' OR 'young people' OR boy* OR girl* OR juvenile*)) OR (AB=(children OR adolescent* OR adolescence OR youth* OR child OR teenager* OR pediatric* OR paediatric* OR kid* OR teen* OR 'young person' OR 'young people' OR boy* OR girl* OR juvenile*))
3	TI ((Anxiety or depression or depressive or "obsessive compulsive disorder" or "OCD" or phobia or phobic or mood disorder or anxiety disorder or panic disorder or agoraphobia or internalising problem* or internalising problem* or internalizing problem* or internalizing disorder*)) OR AB ((Anxiety or depression or depressive or "obsessive compulsive disorder" or "OCD" or phobia or phobic or mood disorder or anxiety disorder or panic disorder or agoraphobia or internalising problem* or internalising problem* or internalizing problem* or internalizing disorder*) OR (MH "Depressive Disorder") OR (MH "Depressive Disorder, Major") OR (MH "Depressive Disorder, Treatment-Resistant") OR (MH "Dysthymic Disorder") OR (MM "Anxiety Disorders+"))
4	(TI=(intervention OR treatment OR therapy OR 'peer support' OR 'support group' OR trial OR psychotherapy OR 'group support' OR 'talking therapy' OR counselling OR 'group therapy' OR 'group intervention' OR 'trauma informed' OR 'self help group' OR 'peer group')) OR (AB=(intervention OR treatment OR therapy OR 'peer support' OR 'support group' OR trial OR psychotherapy OR 'group support' OR 'talking therapy' OR counselling OR 'group therapy' OR 'group intervention' OR 'trauma informed' OR 'self help group' OR 'peer group'))
5	(TI=(stress OR anxiety OR depression OR burnout OR worry* OR 'parent* satisfaction*' OR wellbeing OR 'mental health')) OR (AB=(stress OR anxiety OR depression OR burnout OR worry* OR 'parent* satisfaction*' OR wellbeing OR 'mental health'))
6	#5 AND #4 AND #3 AND #2 AND #1

Database: EMBASE

Search	Terms
1	'parent'/exp OR parent OR 'parents'/exp OR parents OR parental OR 'mother'/exp OR mother OR 'father'/exp OR father OR care*giver OR guardian* OR carer* OR paternal OR 'maternal'/exp OR maternal:ti,ab
2	children OR adolescent* OR adolescence OR youth* OR child OR teenager* OR pediatric* OR paediatric* OR kid* OR teen* OR 'young person' OR 'young people' OR boy* OR girl* OR juvenile*:ti,ab
3	anxiety OR depression OR depressive OR 'obsessive compulsive disorder' OR 'ocd' OR phobia OR phobic OR 'mood disorder' OR 'anxiety disorder':ab,ti OR 'panic disorder' OR agoraphobia OR 'internalising problem*' OR 'internalising disorder*' OR 'internalizing problem*' OR 'internalizing disorder*':ab,ti
4	intervention OR treatment OR therapy OR 'peer support' OR 'support group' OR trial OR psychotherapy OR 'group support' OR 'talking therapy' OR counselling OR 'group therapy' OR 'group intervention' OR 'trauma informed' OR 'self help group' OR 'peer group':ti,ab
5	stress OR anxiety OR depression OR burnout OR worry* OR 'parent* satisfaction*' OR wellbeing OR 'mental health':ti,ab
6	#1 AND #2 AND #3 AND #4 AND #5

For peer review only

Search terms used excluding depression and anxiety terms:

Database: Medline

Search	Term
S1	TI ((parent or parents or parental or mother or father or care*giver or guardian* or carer* or paternal or maternal)) OR AB ((parent or parents or parental or mother or ather or care*giver or guardian* or carer* or paternal or maternal)) OR MM ("Parents+")
S2	TI ((children or adolescent* or adolescence or youth* or child or teenager* or pediatric* or paediatric* or kid* or teen* or young person or young people or boy* or girl* or juvenile*)) OR AB ((children or adolescent* or adolescence or youth* or child or teenager* or pediatric* or paediatric* or kid* or teen* or young person or young people or boy* or girl* or juvenile*)) OR MH ("Child+") OR MM ("Adolescent")
S3	TI ((attention deficit disorder* or “attention deficit hyperactive disorder* “or “ADHD”) OR AB (attention deficit disorder* or “attention deficit hyperactive disorder* “or “ADHD”)OR MH (“Attention Deficit Disorder with Hyperactivity”) OR (TI ((Eating disorder* or eating problem*)) OR AB ((Eating disorder* or eating problem*) OR MH(“Feeding and Eating Disorders+”) OR (TI ((Emerging personality disorder* or emerging personality problem*)) OR AB ((Emerging personality disorder* or emerging personality problem*)) OR MH (“Personality Disorder+”)) OR (TI ((Externalising disorder* or externalising problem* or externalizing disorder* or externalizing problem*) OR AB (Externalising disorder* or externalising problem* or externalizing disorder* or externalizing problem*) OR TX (“Externalising disorder”)) OR (TI ((Oppositional defiant disorder* or oppositional defiant problem*)) OR AB ((Oppositional defiant disorder* or oppositional defiant problem*) OR (MH“Attention Deficit and Disruptive Behavior Disorders+”)) OR (TI ((Psychos* or psychotic disorder* or psychotic problem*)) OR AB ((Psychos* or psychotic disorder* or psychotic problem*)) OR MH (“Psychotic Disorders+)) OR (TI ((Anxiety or depression or depressive or “obsessive compulsive disorder” or “OCD” or phobia or phobic or mood disorder or anxiety disorder or panic disorder or agoraphobia or internalising problem* or internalising problem* or internalizing problem* or internalizing disorder*)) OR AB ((Anxiety or depression or depressive or “obsessive compulsive disorder” or “OCD” or phobia or phobic or mood disorder or anxiety disorder or panic disorder or agoraphobia or internalising problem* or internalising problem* or internalizing problem* or internalizing disorder*) OR (MH "Depressive Disorder") OR (MH "Depressive Disorder, Major") OR (MH "Depressive Disorder, Treatment-Resistant") OR (MH "Dysthymic Disorder") OR (MM "Anxiety Disorders+"))
S4	TI (intervention or treatment or therapy or peer support or support group or trial or psychotherapy or group support or talking therapy or counselling or group therapy or group intervention or trauma informed) OR AB (intervention or treatment or therapy or peer support or support group or trial or psychotherapy or group support or talking therapy or counselling or group therapy or group intervention or trauma informed) OR (MH "Psychotherapy") OR (MH "Self-Help Groups") OR (MH "Peer Group")
S5	TX (stress or anxiety or depression or burnout or worry* or "parent* satisfaction*" or wellbeing or mental health)
S6	TI ((Anxiety or depression or depressive or “obsessive compulsive disorder” or “OCD” or phobia or phobic or mood disorder or anxiety disorder or panic disorder or agoraphobia or internalising problem* or internalising problem* or internalizing problem* or internalizing disorder*)) OR AB ((Anxiety or depression or

	depressive or "obsessive compulsive disorder" or "OCD" or phobia or phobic or mood disorder or anxiety disorder or panic disorder or agoraphobia or internalising problem* or internalising problem* or internalizing problem* or internalizing disorder*) OR (MH "Depressive Disorder") OR (MH "Depressive Disorder, Major") OR (MH "Depressive Disorder, Treatment-Resistant") OR (MH "Dysthymic Disorder") OR (MM "Anxiety Disorders+"))
S6	S1 AND S2 AND S3 AND S4 AND S5 NOT S6

Database: PsycINFO

**Search**	**Term**
S1	TI ((parent or parents or parental or mother or father or care*giver or guardian* or carer* or paternal or maternal)) OR AB ((parent or parents or parental or mother or ather or care*giver or guardian* or carer* or paternal or maternal)) OR MM ("Parents+")
S2	TI ((children or adolescent* or adolescence or youth* or child or teenager* or pediatric* or paediatric* or kid* or teen* or young person or young people or boy* or girl* or juvenile*)) OR AB ((children or adolescent* or adolescence or youth* or child or teenager* or pediatric* or paediatric* or kid* or teen* or young person or young people or boy* or girl* or juvenile*)) OR MH ("Child+") OR MM ("Adolescent")
S3	TI ((attention deficit disorder* or "attention deficit hyperactive disorder* "or "ADHD") OR AB (attention deficit disorder* or "attention deficit hyperactive disorder* "or "ADHD")OR MH ("Attention Deficit Disorder with Hyperactivity") OR (TI ((Eating disorder* or eating problem*)) OR AB ((Eating disorder* or eating problem*) OR MH("Feeding and Eating Disorders+") OR (TI ((Emerging personality disorder* or emerging personality problem*)) OR AB ((Emerging personality disorder* or emerging personality problem*)) OR MH ("Personality Disorder+")) OR (TI ((Externalising disorder* or externalising problem* or externalizing disorder* or externalizing problem*) OR AB (Externalising disorder* or externalising problem* or externalizing disorder* or externalizing problem*) OR TX ("Externalising disorder")) OR (TI ((Oppositional defiant disorder* or oppositional defiant problem*)) OR AB ((Oppositional defiant disorder* or oppositional defiant problem*) OR (MH"Attention Deficit and Disruptive Behavior Disorders+")) OR (TI ((Psychos* or psychotic disorder* or psychotic problem*)) OR AB ((Psychos* or psychotic disorder* or psychotic problem*)) OR MH ("Psychotic Disorders+)) OR (TI ((Anxiety or depression or depressive or "obsessive compulsive disorder" or "OCD" or phobia or phobic or mood disorder or anxiety disorder or panic disorder or agoraphobia or internalising problem* or internalising problem* or internalizing problem* or internalizing disorder*)) OR AB ((Anxiety or depression or depressive or "obsessive compulsive disorder" or "OCD" or phobia or phobic or mood disorder or anxiety disorder or panic disorder or agoraphobia or internalising problem* or internalising problem* or internalizing problem* or internalizing disorder*) OR (MH "Depressive Disorder") OR (MH "Depressive Disorder, Major") OR (MH "Depressive Disorder, Treatment-Resistant") OR (MH "Dysthymic Disorder") OR (MM "Anxiety Disorders+")
S4	TI (intervention or treatment or therapy or peer support or support group or trial or psychotherapy or group support or talking therapy or counselling or group therapy or group intervention or trauma informed) OR AB (intervention or treatment or therapy or peer support or support group or trial or psychotherapy or group support or talking therapy or counselling or group therapy or group intervention or trauma informed) OR (MH "Psychotherapy") OR (MH "Self-Help Groups") OR (MH "Peer Group")

S5	TX (stress or anxiety or depression or burnout or worry* or "parent* satisfaction*" or wellbeing or mental health)
S6	TI ((Anxiety or depression or depressive or “obsessive compulsive disorder” or “OCD” or phobia or phobic or mood disorder or anxiety disorder or panic disorder or agoraphobia or internalising problem* or internalising problem* or internalizing problem* or internalizing disorder*)) OR AB ((Anxiety or depression or depressive or “obsessive compulsive disorder” or “OCD” or phobia or phobic or mood disorder or anxiety disorder or panic disorder or agoraphobia or internalising problem* or internalising problem* or internalizing problem* or internalizing disorder*) OR (MH "Depressive Disorder") OR (MH "Depressive Disorder, Major") OR (MH "Depressive Disorder, Treatment-Resistant") OR (MH "Dysthymic Disorder") OR (MM "Anxiety Disorders+"))
S7	S1 AND S2 AND S3 AND S4 AND S5 NOT S6

Database: CINAHL ULTIMATE

Search	Term
S1	TI ((parent or parents or parental or mother or father or care*giver or guardian* or carer* or paternal or maternal)) OR AB ((parent or parents or parental or mother or ather or care*giver or guardian* or carer* or paternal or maternal)) OR MM ("Parents+")
S2	TI ((children or adolescent* or adolescence or youth* or child or teenager* or pediatric* or paediatric* or kid* or teen* or young person or young people or boy* or girl* or juvenile*)) OR AB ((children or adolescent* or adolescence or youth* or child or teenager* or pediatric* or paediatric* or kid* or teen* or young person or young people or boy* or girl* or juvenile*)) OR MH ("Child+") OR MM ("Adolescent")
S3	TI ((attention deficit disorder* or “attention deficit hyperactive disorder* “or “ADHD”) OR AB (attention deficit disorder* or “attention deficit hyperactive disorder* “or “ADHD”)OR MH (“Attention Deficit Disorder with Hyperactivity”) OR (TI ((Eating disorder* or eating problem*)) OR AB ((Eating disorder* or eating problem*) OR MH(“Feeding and Eating Disorders+”) OR (TI ((Emerging personality disorder* or emerging personality problem*)) OR AB ((Emerging personality disorder* or emerging personality problem*)) OR MH (“Personality Disorder+”)) OR (TI ((Externalising disorder* or externalising problem* or externalizing disorder* or externalizing problem*) OR AB (Externalising disorder* or externalising problem* or externalizing disorder* or externalizing problem*) OR TX (“Externalising disorder”)) OR (TI ((Oppositional defiant disorder* or oppositional defiant problem*)) OR AB ((Oppositional defiant disorder* or oppositional defiant problem*) OR (MH“Attention Deficit and Disruptive Behavior Disorders+”)) OR (TI ((Psychos* or psychotic disorder* or psychotic problem*)) OR AB ((Psychos* or psychotic disorder* or psychotic problem*)) OR MH (“Psychotic Disorders+)) OR (TI ((Anxiety or depression or depressive or “obsessive compulsive disorder” or “OCD” or phobia or phobic or mood disorder or anxiety disorder or panic disorder or agoraphobia or internalising problem* or internalising problem* or internalizing problem* or internalizing disorder*)) OR AB ((Anxiety or depression or depressive or “obsessive compulsive disorder” or “OCD” or phobia or phobic or mood disorder or anxiety disorder or panic disorder or agoraphobia or internalising problem* or internalising problem* or internalizing problem* or internalizing disorder*) OR (MH "Depressive Disorder") OR (MH "Depressive Disorder, Major") OR (MH "Depressive Disorder, Treatment-Resistant") OR (MH "Dysthymic Disorder") OR (MM "Anxiety Disorders+")
S4	TI (intervention or treatment or therapy or peer support or support group or trial or psychotherapy or group support or talking therapy or counselling or group therapy

	or group intervention or trauma informed) OR AB (intervention or treatment or therapy or peer support or support group or trial or psychotherapy or group support or talking therapy or counselling or group therapy or group intervention or trauma informed) OR (MH "Psychotherapy") OR (MH "Self-Help Groups") OR (MH "Peer Group")
S5	TX (stress or anxiety or depression or burnout or worry* or "parent* satisfaction*" or wellbeing or mental health)
S6	TI ((Anxiety or depression or depressive or “obsessive compulsive disorder” or “OCD” or phobia or phobic or mood disorder or anxiety disorder or panic disorder or agoraphobia or internalising problem* or internalising problem* or internalizing problem* or internalizing disorder*)) OR AB ((Anxiety or depression or depressive or “obsessive compulsive disorder” or “OCD” or phobia or phobic or mood disorder or anxiety disorder or panic disorder or agoraphobia or internalising problem* or internalising problem* or internalizing problem* or internalizing disorder*) OR (MH "Depressive Disorder") OR (MH "Depressive Disorder, Major") OR (MH "Depressive Disorder, Treatment-Resistant") OR (MH "Dysthymic Disorder") OR (MM "Anxiety Disorders+"))
S6	S1 AND S2 AND S3 AND S4 AND S5 NOT S6

Database: AMED

Search	Term
S1	TI ((parent or parents or parental or mother or father or care*giver or guardian* or carer* or paternal or maternal)) OR AB ((parent or parents or parental or mother or ather or care*giver or guardian* or carer* or paternal or maternal)) OR MM ("Parents+")
S2	TI ((children or adolescent* or adolescence or youth* or child or teenager* or pediatric* or paediatric* or kid* or teen* or young person or young people or boy* or girl* or juvenile*)) OR AB ((children or adolescent* or adolescence or youth* or child or teenager* or pediatric* or paediatric* or kid* or teen* or young person or young people or boy* or girl* or juvenile*)) OR MH ("Child+") OR MM ("Adolescent")
S3	TI ((attention deficit disorder* or “attention deficit hyperactive disorder* “or “ADHD”) OR AB (attention deficit disorder* or “attention deficit hyperactive disorder* “or “ADHD”)OR MH (“Attention Deficit Disorder with Hyperactivity”) OR (TI ((Eating disorder* or eating problem*)) OR AB ((Eating disorder* or eating problem*) OR MH(“Feeding and Eating Disorders+”) OR (TI ((Emerging personality disorder* or emerging personality problem*)) OR AB ((Emerging personality disorder* or emerging personality problem*)) OR MH (“Personality Disorder+”)) OR (TI ((Externalising disorder* or externalising problem* or externalizing disorder* or externalizing problem*) OR AB (Externalising disorder* or externalising problem* or externalizing disorder* or externalizing problem*) OR TX (“Externalising disorder”)) OR (TI ((Oppositional defiant disorder* or oppositional defiant problem*)) OR AB ((Oppositional defiant disorder* or oppositional defiant problem*) OR (MH“Attention Deficit and Disruptive Behavior Disorders+”)) OR (TI ((Psychos* or psychotic disorder* or psychotic problem*)) OR AB ((Psychos* or psychotic disorder* or psychotic problem*)) OR MH (“Psychotic Disorders+)) OR (TI ((Anxiety or depression or depressive or “obsessive compulsive disorder” or “OCD” or phobia or phobic or mood disorder or anxiety disorder or panic disorder or agoraphobia or internalising problem* or internalising problem* or internalizing problem* or internalizing disorder*)) OR AB ((Anxiety or depression or depressive or “obsessive compulsive disorder” or “OCD” or phobia or phobic or mood disorder or anxiety disorder or panic disorder or agoraphobia or internalising problem* or internalising problem* or internalizing

For peer review only - http://bmjopen.bmj.com/site/about/guidelines.xhtml

	problem* or internalizing disorder*) OR (MH "Depressive Disorder") OR (MH "Depressive Disorder, Major") OR (MH "Depressive Disorder, Treatment-Resistant") OR (MH "Dysthymic Disorder") OR (MM "Anxiety Disorders+")
S4	TI (intervention or treatment or therapy or peer support or support group or trial or psychotherapy or group support or talking therapy or counselling or group therapy or group intervention or trauma informed) OR AB (intervention or treatment or therapy or peer support or support group or trial or psychotherapy or group support or talking therapy or counselling or group therapy or group intervention or trauma informed) OR (MH "Psychotherapy") OR (MH "Self-Help Groups") OR (MH "Peer Group")
S5	TX (stress or anxiety or depression or burnout or worry* or "parent* satisfaction*" or wellbeing or mental health)
S6	TI ((Anxiety or depression or depressive or "obsessive compulsive disorder" or "OCD" or phobia or phobic or mood disorder or anxiety disorder or panic disorder or agoraphobia or internalising problem* or internalising problem* or internalizing problem* or internalizing disorder*)) OR AB ((Anxiety or depression or depressive or "obsessive compulsive disorder" or "OCD" or phobia or phobic or mood disorder or anxiety disorder or panic disorder or agoraphobia or internalising problem* or internalising problem* or internalizing problem* or internalizing disorder*) OR (MH "Depressive Disorder") OR (MH "Depressive Disorder, Major") OR (MH "Depressive Disorder, Treatment-Resistant") OR (MH "Dysthymic Disorder") OR (MM "Anxiety Disorders+"))
S7	S1 AND S2 AND S3 AND S4 AND S5 NOT S6

Database: The Cochrane Library (including the Cochrane Database of Systematic Reviews, the Cochrane Central Register of Controlled Trials (CENTRAL), the Database of Abstracts of Reviews of Effects, the Health Technology Assessment Database, and the NHS Economic Evaluation Database)

**Search**	**Term**
S1	TI ((parent or parents or parental or mother or father or care*giver or guardian* or carer* or paternal or maternal)) OR AB ((parent or parents or parental or mother or ather or care*giver or guardian* or carer* or paternal or maternal)) OR MM ("Parents+")
S2	TI ((children or adolescent* or adolescence or youth* or child or teenager* or pediatric* or paediatric* or kid* or teen* or young person or young people or boy* or girl* or juvenile*)) OR AB ((children or adolescent* or adolescence or youth* or child or teenager* or pediatric* or paediatric* or kid* or teen* or young person or young people or boy* or girl* or juvenile*)) OR MH ("Child+") OR MM ("Adolescent"))
S3	attention deficit disorder* or attention deficit hyperactive disorder* or ADHD or Eating disorder* or eating problem* or Emerging personality disorder or emerging personality problem* or Externalising disorder* or externalising problem* or externalizing disorder* or externalizing problem* or Oppositional defiant disorder* or oppositional defiant problem* or Psychos* or psychotic disorder* or psychotic problem* or Anxiety or depression or depressive or obsessive compulsive disorder or OCD or phobia or phobic or mood disorder or anxiety disorder or panic disorder or agoraphobia or internalising problem* or internalising problem* or internalizing problem* or internalizing disorder*
S4	TI (intervention or treatment or therapy or peer support or support group or trial or psychotherapy or group support or talking therapy or counselling or group therapy or group intervention or trauma informed) OR AB (intervention or treatment or

	therapy or peer support or support group or trial or psychotherapy or group support or talking therapy or counselling or group therapy or group intervention or trauma informed) OR (MH "Psychotherapy") OR (MH "Self-Help Groups") OR (MH "Peer Group")
S5	TX (stress or anxiety or depression or burnout or worry* or "parent* satisfaction*" or wellbeing or mental health)
S6	anxiety OR depression OR depressive OR 'obsessive compulsive disorder' OR 'ocd' OR phobia OR phobic OR 'mood disorder' OR 'anxiety disorder':ab,ti OR 'panic disorder' OR agoraphobia OR 'internalising problem*' OR 'internalising disorder*' OR 'internalizing problem*' OR 'internalizing disorder*':ab,ti
S7	S1 AND S2 AND S3 AND S4 AND S5 NOT S6

Database: Web of Science Core Collection

Search	Terms
1	(TI=(parent OR parents OR parental OR mother OR father OR care*giver OR guardian* OR carer* OR paternal OR maternal)) OR (AB=(parent OR parents OR parental OR mother OR father OR care*giver OR guardian* OR carer* OR paternal OR maternal))
2	(TI=(children OR adolescent* OR adolescence OR youth* OR child OR teenager* OR pediatric* OR paediatric* OR kid* OR teen* OR 'young person' OR 'young people' OR boy* OR girl* OR juvenile*)) OR (AB=(children OR adolescent* OR adolescence OR youth* OR child OR teenager* OR pediatric* OR paediatric* OR kid* OR teen* OR 'young person' OR 'young people' OR boy* OR girl* OR juvenile*))
3	(TI=('attention deficit disorder*' OR 'attention deficit hyperactive disorder*' OR adhd OR 'attention deficit disorder with hyperactivity' OR 'eating disorder*' OR 'eating problem*' OR 'feeding and eating disorders' OR 'emerging personality disorder*' OR 'emerging personality problem*' OR 'personality disorder' OR 'externalising disorder*' OR 'externalising problem*' OR 'externalizing disorder*' OR 'externalizing problem*' OR 'oppositional defiant disorder*' OR 'oppositional defiant problem*' OR 'attention deficit and disruptive behavior disorders' OR psychos* OR 'psychotic disorder*' OR 'psychotic problem*' OR anxiety OR depression OR depressive OR 'obsessive compulsive disorder' OR 'ocd' OR phobia OR phobic OR 'mood disorder' OR 'anxiety disorder' OR 'panic disorder' OR agoraphobia OR 'internalising problem*' OR 'internalising disorder*' OR 'internalizing problem*' OR 'internalizing disorder*)) OR (AB=('attention deficit disorder*' OR 'attention deficit hyperactive disorder*' OR adhd OR 'attention deficit disorder with hyperactivity' OR 'eating disorder*' OR 'eating problem*' OR 'feeding and eating disorders' OR 'emerging personality disorder*' OR 'emerging personality problem*' OR 'personality disorder' OR 'externalising disorder*' OR 'externalising problem*' OR 'externalizing disorder*' OR 'externalizing problem*' OR 'oppositional defiant disorder*' OR 'oppositional defiant problem*' OR 'attention deficit and disruptive behavior disorders' OR psychos* OR 'psychotic disorder*' OR 'psychotic problem*' OR anxiety OR depression OR depressive OR 'obsessive compulsive disorder' OR 'ocd' OR phobia OR phobic OR 'mood disorder' OR 'anxiety disorder' OR 'panic disorder' OR agoraphobia OR 'internalising problem*' OR 'internalising disorder*' OR 'internalizing problem*' OR 'internalizing disorder*))
4	(TI=(intervention OR treatment OR therapy OR 'peer support' OR 'support group' OR trial OR psychotherapy OR 'group support' OR 'talking therapy' OR counselling OR 'group therapy' OR 'group intervention' OR 'trauma informed' OR 'self help group' OR 'peer group')) OR (AB=(intervention OR treatment OR therapy OR 'peer support' OR 'support group' OR trial OR psychotherapy OR 'group support' OR 'talking therapy' OR

	counselling OR 'group therapy' OR 'group intervention' OR 'trauma informed' OR 'self help group' OR 'peer group'))
5	(TI=(stress OR anxiety OR depression OR burnout OR worry* OR 'parent* satisfaction*' OR wellbeing OR 'mental health')) OR (AB=(stress OR anxiety OR depression OR burnout OR worry* OR 'parent* satisfaction*' OR wellbeing OR 'mental health'))
6	#5 AND #4 AND #3 AND #2 AND #1
7	(TI=(anxiety OR depression OR depressive OR 'obsessive compulsive disorder' OR 'ocd' OR phobia OR phobic OR 'mood disorder' OR 'anxiety disorder' OR 'panic disorder' OR agoraphobia OR 'internalising problem*' OR 'internalising disorder*' OR 'internalizing problem*' OR 'internalizing disorder*)) OR (AB=(anxiety OR depression OR depressive OR 'obsessive compulsive disorder' OR 'ocd' OR phobia OR phobic OR 'mood disorder' OR 'anxiety disorder' OR 'panic disorder' OR agoraphobia OR 'internalising problem*' OR 'internalising disorder*' OR 'internalizing problem*' OR 'internalizing disorder*))
8	#6 NOT #7

Database: EMBASE

Search	Terms
1	'parent'/exp OR parent OR 'parents'/exp OR parents OR parental OR 'mother'/exp OR mother OR 'father'/exp OR father OR care*giver OR guardian* OR carer* OR paternal OR 'maternal'/exp OR maternal:ti,ab
2	children OR adolescent* OR adolescence OR youth* OR child OR teenager* OR pediatric* OR paediatric* OR kid* OR teen* OR 'young person' OR 'young people' OR boy* OR girl* OR juvenile*:ti,ab
3	'attention deficit disorder*' OR 'attention deficit hyperactive disorder*' OR adhd OR 'attention deficit disorder with hyperactivity' OR 'eating disorder*' OR 'eating problem*' OR 'feeding and eating disorders' OR 'emerging personality disorder*' OR 'emerging personality problem*' OR 'personality disorder' OR 'externalising disorder*' OR 'externalising problem*' OR 'externalizing disorder*' OR 'externalizing problem*' OR 'oppositional defiant disorder*' OR 'oppositional defiant problem*' OR 'attention deficit and disruptive behavior disorders' OR psychos* OR 'psychotic disorder*' OR 'psychotic problem*' OR anxiety OR depression OR depressive OR 'obsessive compulsive disorder' OR 'ocd' OR phobia OR phobic OR 'mood disorder' OR 'anxiety disorder' OR 'panic disorder' OR agoraphobia OR 'internalising problem*' OR 'internalising disorder*' OR 'internalizing problem*' OR 'internalizing disorder*':ti,ab
4	intervention OR treatment OR therapy OR 'peer support' OR 'support group' OR trial OR psychotherapy OR 'group support' OR 'talking therapy' OR counselling OR 'group therapy' OR 'group intervention' OR 'trauma informed' OR 'self help group' OR 'peer group':ti,ab
5	stress OR anxiety OR depression OR burnout OR worry* OR 'parent* satisfaction*' OR wellbeing OR 'mental health':ti,ab
6	#1 AND #2 AND #3 AND #4 AND #5
7	anxiety OR depression OR depressive OR 'obsessive compulsive disorder' OR 'ocd' OR phobia OR phobic OR 'mood disorder' OR 'anxiety disorder':ab,ti OR 'panic disorder' OR agoraphobia OR 'internalising problem*' OR 'internalising disorder*' OR 'internalizing problem*' OR 'internalizing disorder*':ab,ti
8	#6 NOT #7

PRISMA-P 2015 Checklist

This checklist has been adapted for use with systematic review protocol submissions to BioMed Central journals from Table 3 in Moher D et al: Preferred reporting items for systematic review and meta-analysis protocols (PRISMA-P) 2015 statement. *Systematic Reviews* 2015 **4**:1

An Editorial from the Editors-in-Chief of *Systematic Reviews* details why this checklist was adapted - **Moher D, Stewart L & Shekelle P:** Implementing PRISMA-P: recommendations for prospective authors. *Systematic Reviews* 2016 **5**:15

Section/topic	#	Checklist item	Information reported		Line number(s)
			Yes	No	
**ADMINISTRATIVE INFORMATION**					
**Title**					
Identification	1a	Identify the report as a protocol of a systematic review	✓		1
Update	1b	If the protocol is for an update of a previous systematic review, identify as such		✓	Not applicable
**Registration**	2	If registered, provide the name of the registry (e.g., PROSPERO) and registration number in the Abstract	✓		41-42
**Authors**					
Contact	3a	Provide name, institutional affiliation, and e-mail address of all protocol authors; provide physical mailing address of corresponding author	✓		4-17
Contributions	3b	Describe contributions of protocol authors and identify the guarantor of the review	✓		292-299
**Amendments**	4	If the protocol represents an amendment of a previously completed or published protocol, identify as such and list changes; otherwise, state plan for documenting important protocol amendments		✓	Not applicable
**Support**					
Sources	5a	Indicate sources of financial or other support for the review	✓		301-303
Sponsor	5b	Provide name for the review funder and/or sponsor	✓		301-303
Role of sponsor/funder	5c	Describe roles of funder(s), sponsor(s), and/or institution(s), if any, in developing the protocol		✓	Not applicable
**INTRODUCTION**					
**Rationale**	6	Describe the rationale for the review in the context of what is already known	✓		68-101
**Objectives**	7	Provide an explicit statement of the question(s) the review will address with reference to participants, interventions, comparators, and outcomes (PICO)	✓		98-101
**METHODS**					

For peer review only

BioMed Central The Open Access Publisher

Section/topic	#	Checklist item	Information reported		Line number(s)
			Yes	No	
**Eligibility criteria**	8	Specify the study characteristics (e.g., PICO, study design, setting, time frame) and report characteristics (e.g., years considered, language, publication status) to be used as criteria for eligibility for the review	✓		108-154
**Information sources**	9	Describe all intended information sources (e.g., electronic databases, contact with study authors, trial registers, or other grey literature sources) with planned dates of coverage	✓		156-188
**Search strategy**	10	Present draft of search strategy to be used for at least one electronic database, including planned limits, such that it could be repeated	✓		Supplementary file 1 and 2
STUDY RECORDS					
Data management	11a	Describe the mechanism(s) that will be used to manage records and data throughout the review	✓		203
Selection process	11b	State the process that will be used for selecting studies (e.g., two independent reviewers) through each phase of the review (i.e., screening, eligibility, and inclusion in meta-analysis)	✓		203-208
Data collection process	11c	Describe planned method of extracting data from reports (e.g., piloting forms, done independently, in duplicate), any processes for obtaining and confirming data from investigators	✓		209-238
**Data items**	12	List and define all variables for which data will be sought (e.g., PICO items, funding sources), any pre-planned data assumptions and simplifications	✓		108-154
**Outcomes and prioritization**	13	List and define all outcomes for which data will be sought, including prioritization of main and additional outcomes, with rationale	✓		140-146
**Risk of bias in individual studies**	14	Describe anticipated methods for assessing risk of bias of individual studies, including whether this will be done at the outcome or study level, or both; state how this information will be used in data synthesis	✓		209-238
DATA					
**Synthesis**	15a	Describe criteria under which study data will be quantitatively synthesized	✓		239-276
	15b	If data are appropriate for quantitative synthesis, describe planned summary measures, methods of handling data, and methods of combining data from studies, including any planned exploration of consistency (e.g., I^2, Kendall's tau)	✓		239-276
	15c	Describe any proposed additional analyses (e.g., sensitivity or subgroup analyses, meta-regression)	✓		239-276
	15d	If quantitative synthesis is not appropriate, describe the type of summary planned	✓		239-276
**Meta-bias(es)**	16	Specify any planned assessment of meta-bias(es) (e.g., publication bias across studies, selective reporting within studies)	✓		254-256
**Confidence in cumulative evidence**	17	Describe how the strength of the body of evidence will be assessed (e.g., GRADE)	✓		209-238

BioMed Central
The Open Access Publisher

For peer review only - http://bmjopen.bmj.com/site/about/guidelines.xhtml
